# Machine learning predicts individual cancer patient responses to therapeutic drugs with high accuracy

**DOI:** 10.1038/s41598-018-34753-5

**Published:** 2018-11-06

**Authors:** Cai Huang, Evan A. Clayton, Lilya V. Matyunina, L. DeEtte McDonald, Benedict B. Benigno, Fredrik Vannberg, John F. McDonald

**Affiliations:** 1 School of Biological Sciences and Petit Institute for Bioengineering and Bioscience, Georgia Institute of Technology, 315 Ferst Drive, Atlanta, GA 30332 USA; 2 Integrated Cancer Research Center, Georgia Institute of Technology, 315 Ferst Drive, Atlanta, GA 30332 USA; 3 Ovarian Cancer Institute, 960 Johnson Ferry Road, Atlanta, GA 30342 USA

## Abstract

Precision or personalized cancer medicine is a clinical approach that strives to customize therapies based upon the genomic profiles of individual patient tumors. Machine learning (ML) is a computational method particularly suited to the establishment of predictive models of drug response based on genomic profiles of targeted cells. We report here on the application of our previously established open-source support vector machine (SVM)-based algorithm to predict the responses of 175 individual cancer patients to a variety of standard-of-care chemotherapeutic drugs from the gene-expression profiles (RNA-seq or microarray) of individual patient tumors. The models were found to predict patient responses with >80% accuracy. The high PPV of our algorithms across multiple drugs suggests a potential clinical utility of our approach, particularly with respect to the identification of promising second-line treatments for patients failing standard-of-care first-line therapies.

## Introduction

A primary goal of precision cancer medicine is the accurate prediction of optimal drug therapies based upon the personalized molecular profiles of patient tumors^1^. Ideally, such predictions are based upon well-established molecular cause-and-effect relationships that are disrupted in cancer cells. A notable example is the targeted inhibition of the *Abl* tyrosine kinase gene in the treatment of chronic myelogenous leukemia (CML)^2^. Unfortunately, the molecular processes underlying most cancers, and especially solid tumors, are currently not as well understood as for CML^3^. An alternative path to accurate predictions is based simply on observed, significant correlations, even when the underlying causal connections are unknown or incompletely understood.

The foundation of accurate correlative predictions is built upon extensive and reliable bodies of data, and the volume of cancer-relevant data being generated and computationally stored on a daily basis vastly exceeds what could be even imagined only a few decades ago. For example, the volume of cancer-relevant molecular data being generated by genomic studies alone (DNA sequencing, RNA expression, *etc*.) is currently doubling about every 6-7 months and, within the next decade, is estimated to constitute up to 40 million gigabytes a year^4^.

The search for significant correlations in cancer-relevant datasets is a task ideally suited to computers and specifically to a branch of artificial intelligence called machine learning (ML). Toward that end, a number of ML-based approaches have been developed in recent years that input the genomic profiles of individual patient tumors and output predictions of optimal drug responses based upon correlations embedded within previously established datasets^5^. We recently introduced an “open source” support vector machine (SVM)-based algorithm that inputs gene expression profiles of cancer cells to predict the response of individual cancers to chemotherapeutic drugs^6^. In addition to accurately predicting the response of a variety of cancer cell lines to 7 commonly prescribed chemotherapeutic drugs, we employed the algorithm to predict the sensitivities of 273 ovarian cancer patients to these same drugs^6^. These predictions were shown to correlate significantly with previously reported average response rates of independent groups of ovarian cancer patients to these drugs (Linear regression p value = 0.0031, R^2^ = 0.8201) lending further credibility to our approach^6^.

While the importance of the initial testing of drug prediction algorithms in well-characterized cancer cell lines cannot be overstated, eventual adoption of this computational approach into clinical practice will require extensive testing in human cancer patients. Toward this end, we report here an initial series of studies designed to evaluate the accuracy of our SVM-based algorithms to predict the responses of individual cancer patients to a variety of standard-of-care chemotherapeutic drugs from gene-expression profiles (RNA-seq or microarray) of individual patient tumors. The accuracies of the models to predict responses to a variety of drugs across 175 patients ranged from 81.5% to 82.6%. The potential clinical utility of our SVM-based approach, particularly with respect to the selection of drugs for patients resistant to first-line chemotherapies, is discussed.

## Results

### The response of individual cancer patients to gemcitabine or 5-fluorouracil therapy is predicted with >80% accuracy

To assess the accuracy of our SVM-based algorithms to predict drug response on an individual patient basis, we first employed matched sets of gene-expression and drug-response profiles from The Cancer Genome Atlas (TCGA) database^7^. The TCGA database is comprised of 2.5 petabytes of data including the genomic profiles of tumor and matched normal tissues from more than 11,000 patients representing 33 types of human cancers. Despite the impressive size of this dataset, we were limited because we require not only gene-expression profiles of patient tissues but detailed information on each patient’s individual response to chemotherapy as well. Since the availability of such correlated sets of data for specific cancer types is currently limited, we combined TCGA data of patients associated with a diversity of cancer types but for which the response profiles to two commonly employed chemotherapeutic agents, gemcitabine (GEM) and 5-fluorouracil (5-FU), have been well documented. In this way, we were able to establish a dataset comprised of expression profiles (RNA-seq) and drug response profiles of 152 patients (92 treated with gemcitabine, 60 treated with 5-fluorouracil) (Supplementary Table S1).

Independent predictive models were built for GEM and for 5-FU utilizing the gene expression and patient outcome data obtained from the TCGA database. Unlike our earlier models that were built using microarray gene-expression data^6^, the gene-expression values in the TCGA dataset are recorded as RNA-seq profiles^8^. Our model building and testing methods, however, remain essentially as previously described^6^. The standard normalization procedures are described in the Methods section.

In the TCGA database, patient responses to drugs are grouped into 4 categories: complete response, partial response, progressive disease and stable disease. Since the current configuration of our algorithms require a binary input with respect to drug response, we classified patients displaying either complete or partial response to the drug treatment as responders (R) and those displaying progressive or stable disease following treatment as non-responders (NR) (Supplementary Table S2).

The profiles of 75% of the patients (*i.e*., 69 patients for GEM; 45 patients for 5-FU) were randomly selected to establish the learning datasets for model building and the remaining 25% (*i.e*., 23 patients for GEM; 15 patients for 5-FU) were employed as the test datasets for initial evaluation of the models.

**Figure 1:**
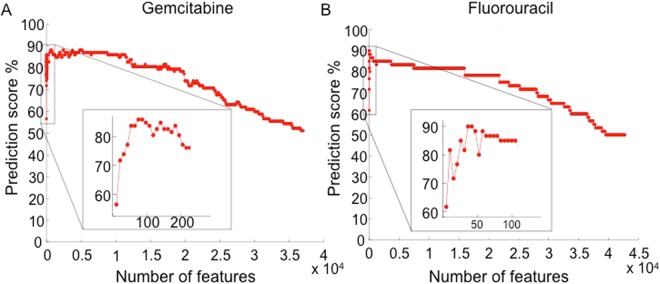
Evolution of accuracy of predicted response to gemcitabine (**A**) and 5-fluorouracil (**B**) using SVM-RFE selection for gene classifiers.

ML models built from large datasets typically contain uninformative features that can reduce predictive accuracy. For this reason, several feature selection methods have been developed to establish subsets of features with optimal predictive accuracy^9,10^. In our studies, we employ a recursive feature elimination (RFE) method^6^ to select for features (*i.e*., gene-expression patterns) that can optimally distinguish between responders and non-responders. To begin, the least relevant features of the model from the sorted feature list (Supplementary Table S3) are discarded, as previously described^6^. Fig. 1 depicts the evolution of predictive accuracy using SVM-RFE feature selection for increased sensitivity to GEM and 5-FU. The minimum number of informative features associated with optimally predicted responsiveness to GEM was 81 and for 5-FU was 31. Although the majority of these genes remain functionally unannotated, a number have either directly or indirectly been previously associated with apoptosis, which is consistent with the DNA damaging action of both of these drugs (Supplementary Table S4).

**Figure 2:**
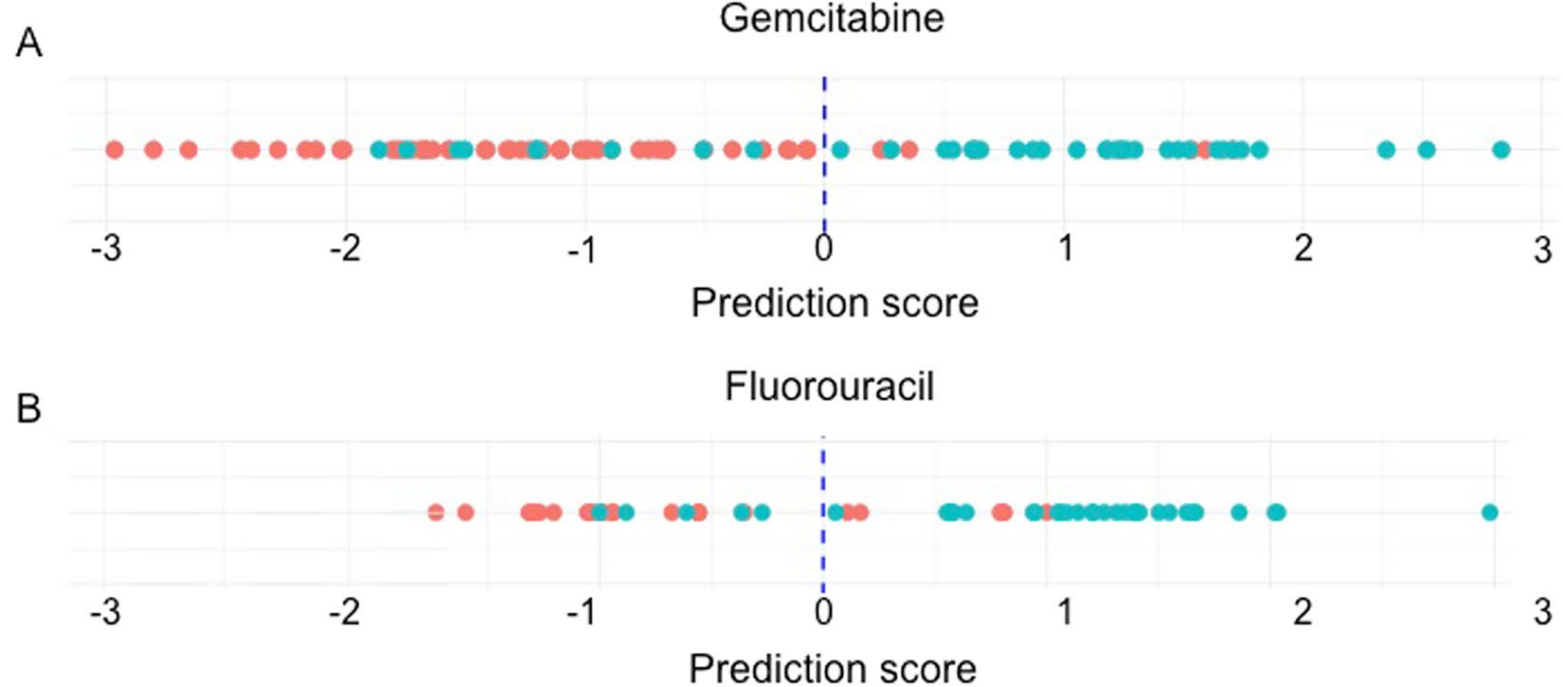
Individual prediction of response to chemotherapeutic drugs. The SVM algorithms output binary classifications for gemcitabine and 5-fluorouracil (red = observed drug non-responder; blue = observed drug responder) established through a decision function that numerically separates tumors predicted to respond to the drug (positive score) from those predicted to be non-responders (negative score).

Employing a set of most informative features, we generated drug prediction scores for each patient. Scores greater than “0” indicate a predicted positive response to the drug while scores less than “0” are predictive of drug resistance. Fig. 2 displays the distribution of prediction scores for the 92 patients treated with gemcitabine and the 60 patients treated with 5-FU (see also Supplementary Table S2). Patients observed to respond positively to the drug therapy are represented in the figures by blue dots and those observed not to respond to the therapy by red dots. The overall accuracies (GEM 81.5%; 5-FU 81.7%; PPV GEM 77.8%; 5-FU 83.3%; NPV GEM 83.9%; 5-FU 79.2%), sensitivities (GEM 75.7%; 5-FU 85.7%), and specificities (GEM 85.5; 5-FU 76.0%) of the two models were determined by leave-one-out cross validation (LOOCV) as previously described^6^. The high accuracy of our SVM-based models to predict individual patient responses to these two chemotherapeutic drugs is comparable to our previously reported accuracy (>80%) to predict the collective responses of 273 ovarian cancer patients to seven chemotherapeutic drugs^6^.

### The response of individual ovarian cancer patients to standard-of-care therapies is predicted with high accuracy

The above studies generally support the potential of our SVM-RFE approach to accurately predict the drug responsiveness of individual cancer patients. To further assess the accuracy and evaluate the potential clinical usefulness of our approach, we conducted gene-expression profiling of tumors collected from a randomly selected group of ovarian cancer patients and used SVM-RFE-based models to predict patient responsiveness to eight drugs often used in the treatment of ovarian cancer (carboplatin, cisplatin, paclitaxel, docetaxel, gemcitabine, doxorubicin, gefitinib, and topotecan).

Samples of primary tumors collected from 23 ovarian cancer patients (Supplementary Table S5) were snap frozen in liquid nitrogen within one minute of surgical removal and transferred to the lab for laser capture microdissection of cancer cells and subsequent microarray gene-expression analysis (Affymetrix, U133 Plus 2.0 arrays) as previously described^11^. Nearly all (21/23) of the collected samples were serous papillary ovarian cancers with the remaining two classified as an adenocarcinoma and a malignant mesodermal mixed tumor (MMMT). The vast majority (19/23) of the samples were derived from patients with moderate to high-grade (Grade 2-3), late stage (Stage III/IV) disease. Four of the samples were derived from patients with high-grade early-stage disease (Stage I/II).

The majority of patients (17/23) were administered chemotherapy shortly after de-bulking surgery with six patients receiving neo-adjuvant chemotherapeutic treatment prior to surgery. Most of the patients were treated with standard-of-care carboplatin/paclitaxel combination therapy (18/23). One patient was treated with carboplatin and gemcitabine, one with carboplatin and docetaxel and one with carboplatin, cisplatin and paclitaxel combination therapies. Only two patients were treated with a single drug-one with topotecan and one with doxorubicin (Table 1).

**Table 1:** Predicted and observed responses of 23 ovarian cancer patients treated with one or more of eight chemotherapeutic drugs.

Patient	Drug	Observed Response	Predicted	Predicted	Predicted	Predicted	Predicted	Predicted	Predicted	Predicted
			Carboplatin	Paclitaxel	Cisplatin	Gemcitabine	Docetaxel	Doxorubicin	Gefitinib	Topotecan
229	Carbo&GEM	R (TP)	NR (FN)	NR	R	R (TP)	R	NR	NR	NR
242	Carbo&Taxol	R (TP)	R (TP)	NR (FN)	NR	NR	R	R	R	NR
272	Carbo&Taxol	NR (FP)	NR (TN)	R (FP)	NR	R	R	NR	NR	NR
286	Carbo&Taxol	NR (TN)	NR (TN)	NR (TN)	NR	NR	NR	R	R	NR
317	Carbo&Taxol	R (TP)	R (TP)	R (TP)	R	R	NR	R	NR	NR
336	Carbo&Taxol	R (TP)	R (TP)	R (TP)	R	R	R	NR	NR	NR
367	Carbo&Taxol	R (TP)	R (TP)	R (TP)	NR	R	NR	NR	NR	NR
413	Carbo&Taxol	R (TP)	R (TP)	R (TP)	NR	R	R	R	NR	NR
489	Carbo&Taxol	R (TP)	R (TP)	R (TP)	NR	NR	NR	NR	NR	R
528	Carbo&Taxol	R (TP)	R (TP)	R (TP)	NR	NR	R	NR	NR	NR
542	Carbo&Taxol	R (TP)	R (TP)	R (TP)	NR	R	NR	NR	NR	NR
545	Carbo&Taxol	NR (TN)	NR (TN)	NR (TN)	NR	R	R	NR	NR	R
588	Carbo&Taxol	R (TP)	R (TP)	NR (FN)	R	R	R	NR	NR	R
617	Carbo&Taxol	R (TP)	R (TP)	R (TP)	NR	R	NR	NR	NR	NR
620	Carbo&Taxol	R (TP)	R (TP)	R (TP)	NR	R	NR	NR	NR	NR
813	Carbo/Cis/Taxol	R (TP)	NR (FN)	R (TP)	R (TP)	NR	NR	NR	NR	NR
992	Topotecan	NR(TN)	R	NR	NR	R	NR	NR	NR	NR (TN)
1012	Carbo & docetaxel	R(TP)	NR (FN)	NR	R	R	R (TP)	R	NR	NR
1122	Carbo&Taxol	R (TP)	NR (FN)	R (TP)	NR	R	NR	NR	NR	NR
1129	Doxorubicin	R (TP)	NR	R	R	R	NR	R (TP)	NR	NR
1145	Carbo&Taxol	NR (FP)	R (FP)	NR (TN)	NR	NR	NR	NR	NR	R
BJ1	Carbo&Taxol	R (FN)	NR (FN)	NR (FN)	R	R	NR	NR	NR	NR
BJ4	Carbo&Taxol	R (TP)	NR (FN)	R (TP)	R	R	R	R	NR	NR
Totals:		17TP,2TN,3FP,1FN								

**Figure 3:**
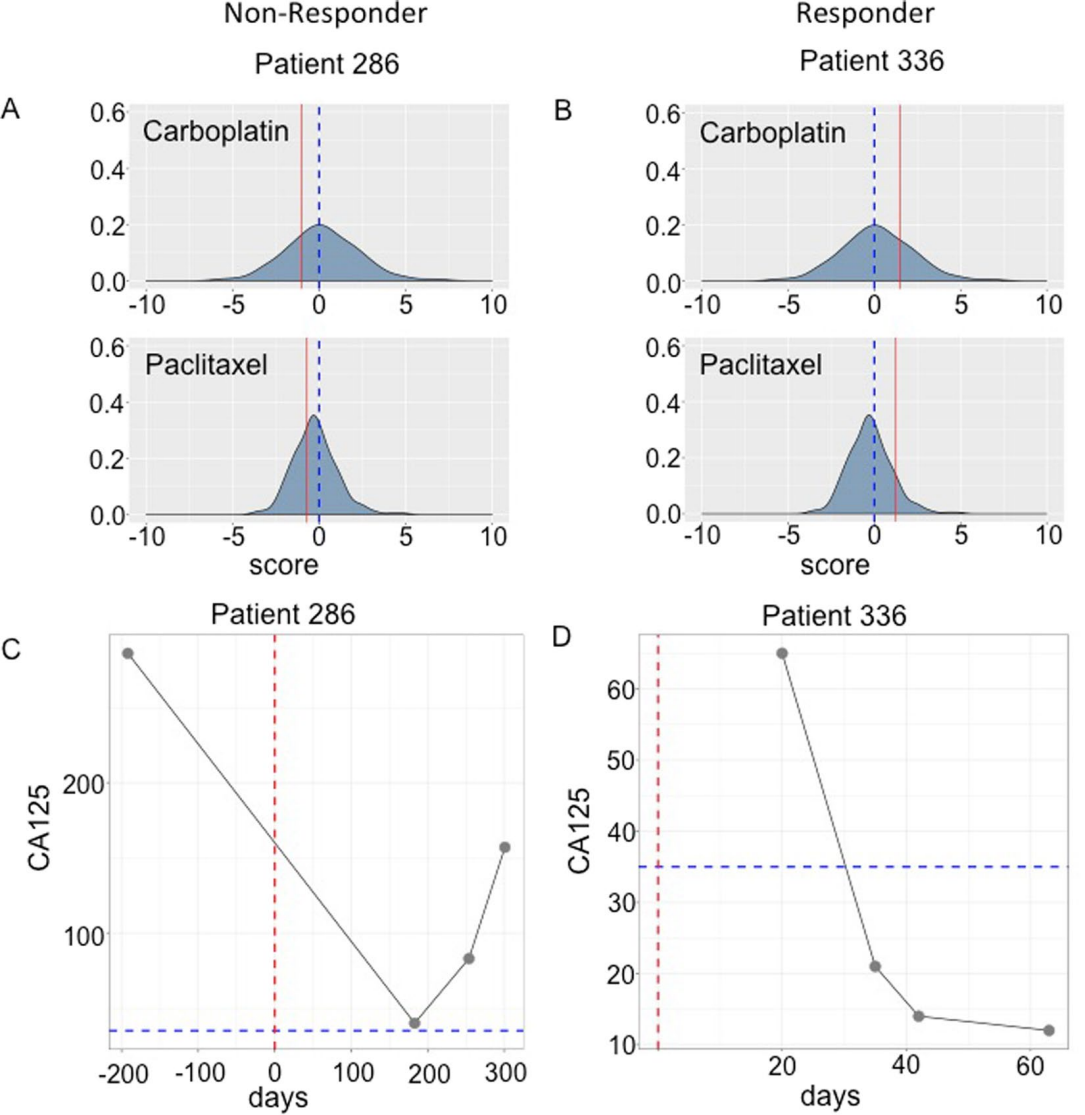
Comparison of the predicted and observed responses of two ovarian cancer patients to carboplatin and paclitaxel therapies. The predicted response scores of each patient (red line) are plotted over the distribution of the previously predicted scores of 273 ovarian cancer patients^6^. Patient 286 (**A**) is predicted not to respond to either drug (negative scores) while patient 336 (**B**) is predicted to respond to both. (**C**,**D**) Patients are considered to be responsive to treatments if their respective CA-125 values dropped below normal values (<35, dashed blue line; dashed red line = day of surgery). Patient 286 (**C**) is a non-responder while patient 336 (**D**) is a responder.

The RNA expression profiles of significantly expressed genes were uploaded to our previously established SVM-algorithms^12^ to generate drug prediction scores for each of eight chemotherapeutic drugs. We included all microarray probe sets for each gene in our analysis because, as previously demonstrated^6^, the averaging of expression values over multiple probe sets can significantly reduce predictive accuracies. As described above, the predictive algorithms generate scores for each drug. Scores greater than “0” indicate a predicted positive response to the drug while scores less than “0” are predictive of drug resistance (*e*.*g*., Fig. 3A and B; and Supplementary Fig. S1).

The majority of the 23 ovarian cancer patients analyzed were predicted to respond favorably to gemcitabine (17/23), paclitaxel (14/23) and carboplatin (13/23) (Table 1), with less than half to cisplatin (9/23) and docetaxel (10/23). Less than a third of the 23 patients were predicted to respond to doxorubicin (7/23), topotecan (4/23) or gefitinib (2/23). These predicted efficacies are generally consistent with our earlier group predictions of 273 OC patients. The slight inconsistencies may be attributable to sampling error due to the relatively few patients employed in the current study.

To evaluate the accuracy of our predictions, patient responses to administered chemotherapies were monitored by measurement of CA-125 values prior and subsequent to treatment. Patients were considered to be responsive to treatments if their respective CA-125 values dropped below normal values (<35) (Fig. 3C and D; and Supplementary Fig. S2).

Our algorithms predict responses to individual drugs and in those few cases where patients were treated with a single drug, evaluation of the model’s predictive accuracy is straightforward (*e.g*., patients 992 and 1129; Table 1). However, standard-of-care chemotherapy for ovarian cancer patients typically involves treatment with multiple drugs, most commonly, carboplatin and paclitaxel. In those cases where patients were observed to positively respond to the combination therapies, the prediction was scored as “true positive” (TP) if the patient is predicted to respond to at least one of the administered drugs (*e.g*., patients 242, 367 and 588). Conversely, in cases where patients were observed to not respond to the combination therapy, the prediction was scored as “false positive” (FP) if the patient was predicted to respond to at least one of the drugs (*e.g*., patients 272 and 1145). Instances where the patient is both predicted and observed not to respond to the combination therapy are scored as “true negative” (TN) (*e.g*., patients 286 and 545) while cases where the patient responded to the combination therapy but is predicted not to respond to any of the administered drugs was scored as “false negative” (FN) (*e.g*., patient BJ1; Table 1).

Based on these criteria, the computational predictions resulted in 17 TP, 2 TN, 3 FP and 1 FN. This equates to a positive predictive value (PPV) of 85% (sensitivity 94.4%), the negative predictive value (NPV) was 66.7% (specificity of 40%) equating to an overall accuracy of 82.6%. The low specificity may, in part, be due to sampling error since only five patients were observed to be non-responders by the above criteria in this study group.

**Figure 4:**
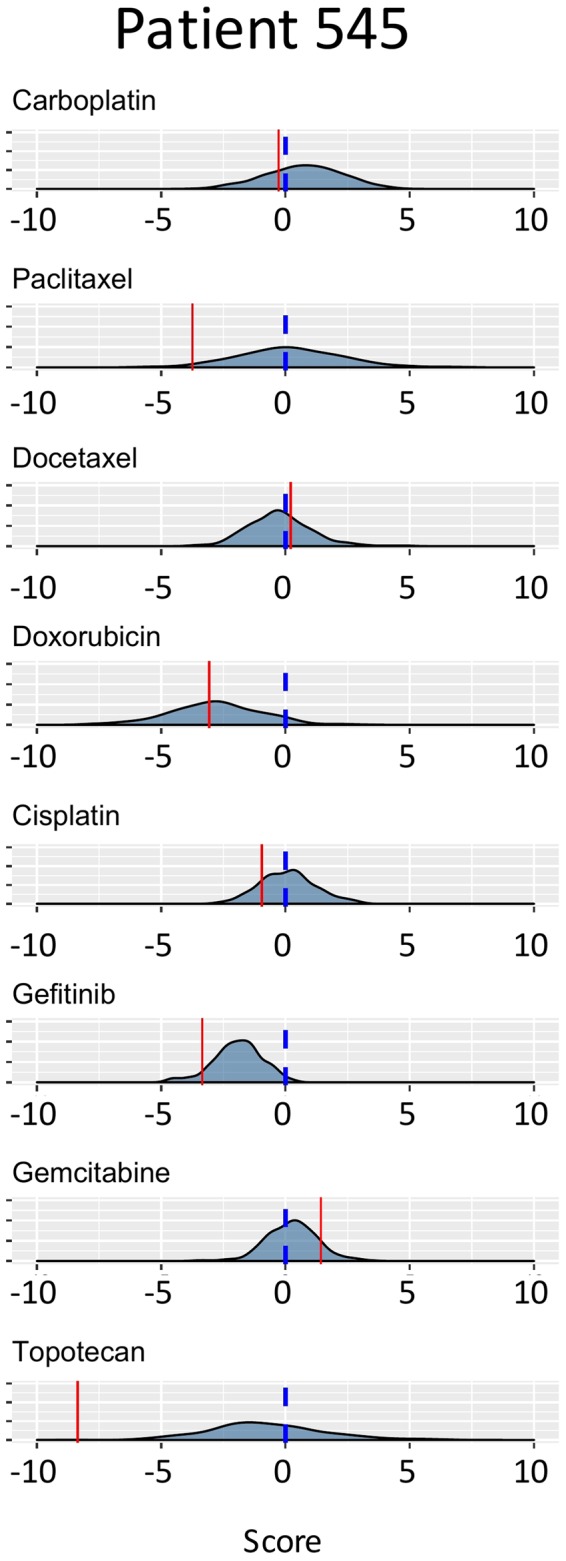
Algorithms with high positive predictive value (PPV) may be of particular clinical benefit in the selection of alternative second-line chemotherapies. Patient 545 was predicted (and observed, see Table 1) not to respond to standard-of-care carboplatin/paclitaxel therapy. Of possible second-line therapies, gemcitabine is predicted to be the preferred choice.

One possible clinically useful application of our models is depicted in Fig. 4. As shown (see also Supplementary Fig. S1), the predictive scores of an individual patient can be mapped across the distributed scores of all previously profiled patients providing information on those drugs most likely to be effective as treatments for an individual patient. Patient 545 was both predicted and observed (Table 1) not to respond to carboplatin/paclitaxel treatment. An estimated 20–30% of all ovarian cancer patients treated with this standard-of-care combination therapy similarly fail to respond to treatment^13^ leaving physicians with the decision as to what to try next. ML-based models with validated high positive predictive values, such as reported here, may provide physicians with a useful alternative to the traditional trial-and-error strategies. For example, based on the predicted responses of patient 545 to the possible second-line drugs modeled in this study, gemcitabine stands out as a preferred choice.

## Discussion

Cancer is a complex disease. The fact that there are a multitude of possible molecular paths to developing even the same type of cancer explains, in large measure, why the response to any given chemotherapeutic drug can be highly variable across patients^14^. Our increasing ability to accurately profile individual patient tumors on the molecular level is widely viewed as a promising resolution to this problem. Indeed, a major goal of modern cancer medicine is the ability to accurately predict optimal drug therapies based upon the personalized molecular profiles of individual patient tumors.

Accurate predictions in cancer biology, as in all areas of science, can be based upon established cause-and-effect relationships or upon significant correlations detected in large sets of relevant data. While we are well on our way to the day when we may fully understand the molecular causes of all cancers and treat them accordingly, we are not there yet. One promising interim solution is the application of prediction algorithms derived from ML-detected correlations between the molecular profiles of large numbers of cancers and associated responses to a variety of therapeutic drugs^15^.

We recently reported on the use of our open access SVM-based algorithms to accurately (>80%) predict the collective response of 273 ovarian cancer patients to seven commonly prescribed chemotherapeutic drugs^6^. In this current study, we were interested in evaluating the performance of our approach to predict individual patient responses to drugs based on gene expression profiles of each individual’s tumor. Employing gene expression (RNA-seq) profiles of 152 cancer patients downloaded from the TCGA database, we were able to predict the response of individual patients treated with either gemcitabine or 5-FU with >81% accuracy. In a second study, the response of individual ovarian cancer patients to eight commonly prescribed chemotherapeutic drugs, based on microarray gene expression profiles of each patient’s tumor, was predicted with an overall accuracy of 83% and a PPV of 85%. The high PPV of our algorithms across multiple drugs suggests a potential clinical utility of our approach to identify promising second-line treatments for patients failing standard-of-care first-line therapies.

It should be noted that although our models have, thus far, focused on the predicted response of cancer patients to current standard-of-care drug therapies for which sufficient datasets are available, the approach is equally as well applicable to emerging immuno- and other targeted gene therapies where patient responses are also known to be variable and likely dependent upon the personalized genomic makeup of individual tumors e.g., ^16^.

## Methods

### TCGA data

Clinical trial data (12,051 records) were downloaded from TCGA for 32 types of cancer. Drug response information for each patient was obtained by querying the TCGA clinical cases field via the Genomic Data Common’s (GDC) API. The clinical data needed to be assessed to determine which were the best drugs to investigate. We first defined a “responder” as a patient who had partial or complete response and a “non-responder” as a patient with clinically progressive or stable disease. To determine viable candidate drugs for the analysis, we required a sample size of at least 30 patients for the drug of interest with at least 15 for each type of response. The clinical trial data were then cleaned using fuzzy matching and manual curation to ensure consistency of drug name and formatting, and only patients on a single candidate drug at a time were retained for evaluation. From this list, gemcitabine and fluorouracil were selected as optimal drugs for our analysis.

Corresponding upper-quartile normalized, fragments per kilobase of transcript per million mapped reads (UQ-FPKMs) from patient primary tumor samples were downloaded using the GDC API. GDC provides UQ-FPKMs to facilitate cross-sample comparison and differential expression analysis. This RNA-seq based expression normalization method can be defined as:

$${\rm{FPKM}}=[{{\rm{RM}}}_{{\rm{g}}}\ast {10}^{9}]/[{{\rm{RM}}}_{{\rm{75}}}\ast {\rm{L}}]$$

- RM_g_: The number of reads mapped to the gene.
- RM_75_: The number of reads mapped to the 75^th^ percentile gene in the alignment.
- L: The length of the gene in base pairs.

Each UQ-FPKM value is analyzed as a separate feature for each sample. Genes were removed from the dataset if >25% of the samples displayed a zero expression value. We applied SVM on training data to get weights for each feature, and sorted the features based on the weights (Supplementary Table S3).

Linear support vector machine (SVM) is employed recursively as a classification model to separate samples into two classes (drug sensitive and drug resistant) as described previously^6^. Briefly, the samples are represented as a vector *x*, and the two classes are divided in the dataspace by a hyperplane *wx′* + *b* = 0 that maximizes the margins between the learning samples of the two classes. This margin is defined such that:

$$wx^{\prime} +b\ge 1,c=1$$

$$wx^{\prime} +b\le -\,1,c=-\,1$$

The test prediction is a binary classification and the prediction scores for test samples are generated using the decision function:

$$prediction\_score=-\,1x(\sum _{f=1}^{i}{w}_{f}{x}_{f}+b)$$

where *w* and *b* are respectively the weight vector and bias parameters from the SVM model. The normalized test sample gene-expression data are the input *x* with RFE selected *i* number of features. We call a sample drug sensitive if the computed score is higher than 0, and drug resistant if the score is lower than 0.

Recursive feature elimination (RFE) is employed to determine the minimum set of features that maximize accuracy on the test dataset. The approach starts by removing the 100 features with the lowest ranked weights in the sorted feature list. An SVM model is subsequently built using the remaining features and this process proceeds recursively until the number of remaining features reaches 100. Thereafter, features are removed one at a time until the most informative set of features is obtained. If multiple highest accuracy models are generated, the model with the fewest number of features is adopted. The final predictive model for each drug is the one with the most informative set of features. Leave one out cross-validation (LOOCV) is subsequently used to evaluate the performance of each of the models as previously described^6^.

### Ovarian cancer data

Informed patient consents were obtained under appropriate Georgia Institute of Technology Institutional Review Board protocol (H14337). Samples of primary tumors collected from 23 ovarian cancer patients (Supplementary Table S5) at Northside Hospital (Atlanta) were snap frozen in liquid nitrogen within one minute of surgical removal and transferred to the lab for laser capture microdissection of cancer cells and subsequent microarray gene-expression analysis (Affymetrix, U133Plus 2.0 arrays, ThermoFisher Scientific) as previously described^11^. Individual gene expression microarray (.CEL) files were normalized one by one against the original NCI 60 gene expression microarray data specific to each array (both Affymetrix U133 Plus 2 and Human Exon Array) using standard quantile normalization and using the mean of each probe. This approach creates distributions for each array that are as similar as possible in terms of statistical properties.

Patient responses to administered chemotherapies were monitored by measurement of CA-125 values prior and subsequent to treatment. Patients were considered to be responsive to treatments if their respective CA-125 values dropped below normal values (<35) (Fig. 3C and D; and Supplementary Fig. S2).

Microarray gene expression and patient drug response data were uploaded to our predictive algorithms^8^ and predictions were generated as previously described^6^.

## Electronic supplementary material

Supplementary Information

Supplementary Dataset

## Data Availability

The ovarian microarray datasets analyzed during the current study are available in the Gene Expression Omnibus (GSE38666, GSE GSE112798); data used from TCGA Research Network are available: https://docs.gdc.cancer.gov/Data/Bioinformatics_Pipelines/Expression_mRNA_Pipeline/.
