# Supplementary material for: Machine learning predicts individual cancer patient responses to therapeutic drugs with high accuracy
Source: Sci Rep. 2018 Nov 6;8:16444. doi: 10.1038/s41598-018-34753-5 (PMC6219522; doi:10.1038/s41598-018-34753-5)
Supplement: Supplementary file 1 — Supplementary Information [file 41598_2018_34753_MOESM1_ESM.pdf]

Supplementary Information for:

Machine learning predicts individual cancer patient responses to therapeutic drugs with high accuracy

Cai Huang[1], Evan A. Clayton[1], Lilya V. Matyunina[1], L. DeEtte McDonald[1], Benedict B. Benigno[2,3,] Fredrik Vannberg[1,2,‡], and John F. McDonald[1,2,3,‡, *]

[1]School of Biological Sciences and Petit Institute for Bioengineering and Bioscience, Georgia Institute of Technology, 315 Ferst Drive, Atlanta, GA 30332, USA.

[2]Integrated Cancer Research Center, Georgia Institute of Technology, 315 Ferst Drive, Atlanta, GA 30332, USA.

[3]Ovarian Cancer Institute, 960 Johnson Ferry Road, Atlanta, GA, 30342, USA.

‡ these authors contributed equally to the work

*To whom correspondence should be addressed:

John F. McDonald, Georgia Institute of Technology, 315 Ferst Drive, Atlanta, GA, 30332, USA;

Phone: 404-385-6630; FAX: 404-894-2291; email: john.mcdonald@biology.gatech.edu

Supplementary Fig. S1. The predicted response scores of each of the 23 ovarian cancer patients analyzed in this study (red lines) are plotted over the distribution of the previously predicted scores of 273 ovarian cancer patients[6] for eight chemotherapeutic drugs.

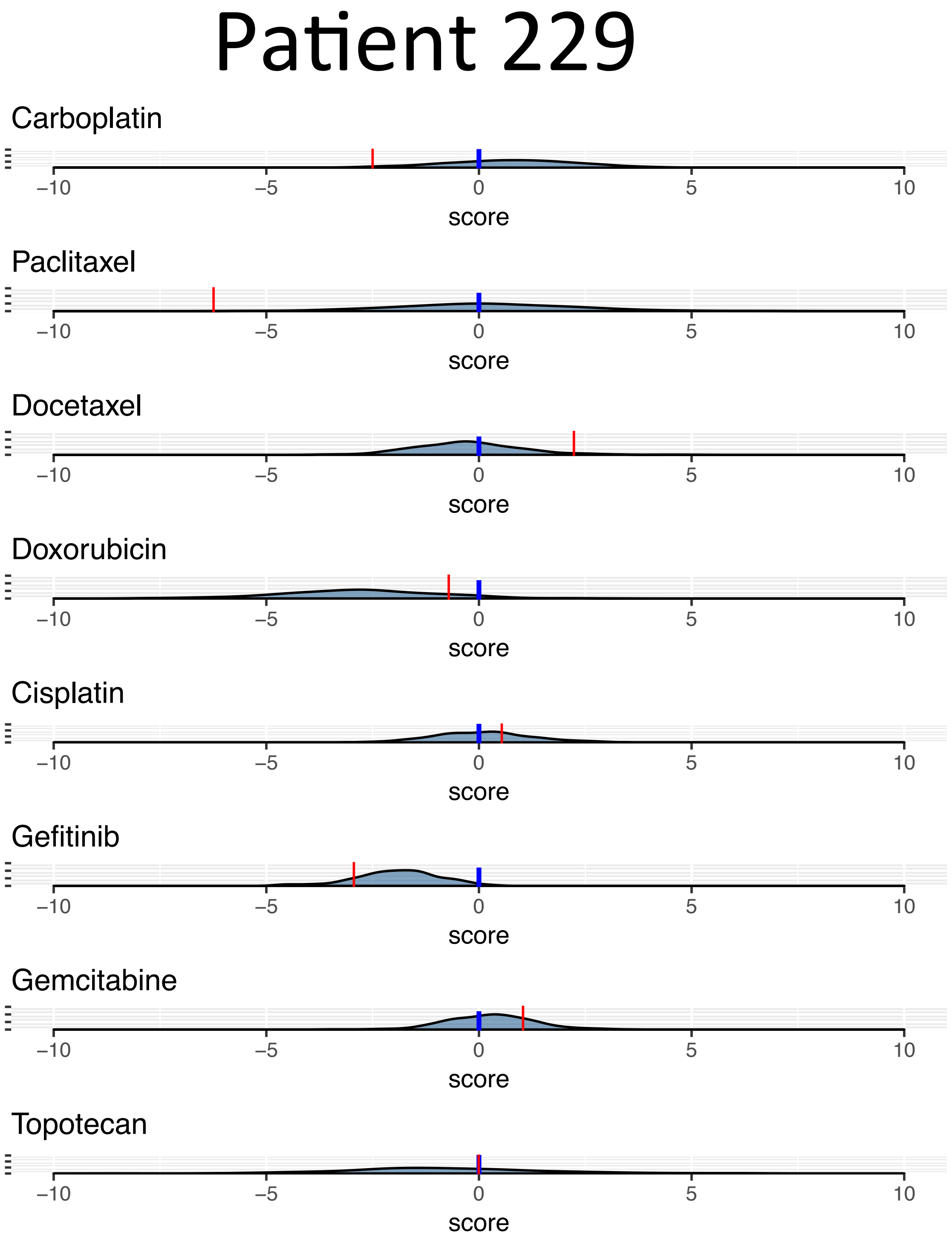

Patient 242

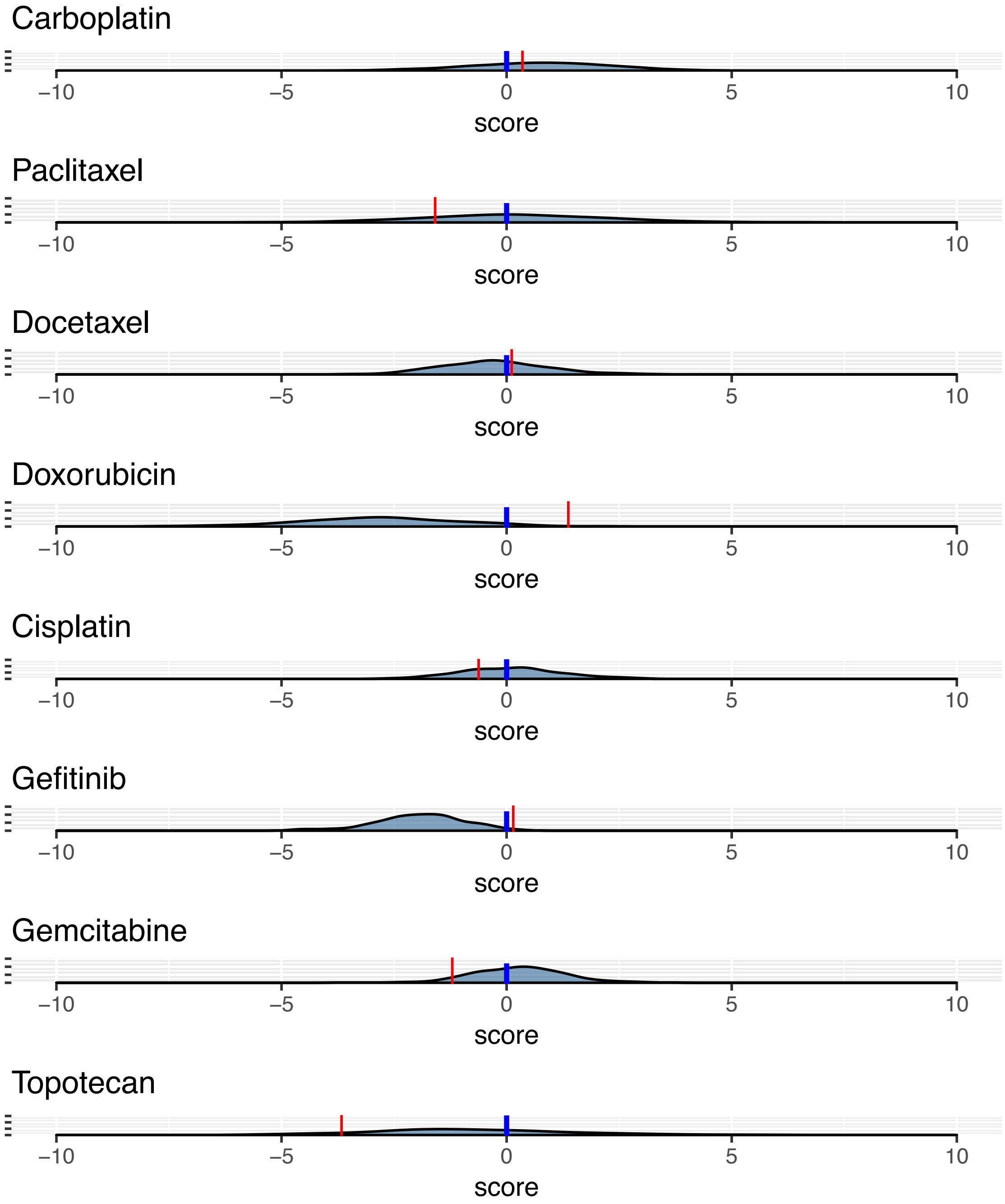
Carboplatin
−10 −5 0 5 10
score
Paclitaxel
−10 −5 0 5 10
score
Docetaxel
−10 −5 0 5 10
score
Doxorubicin
−10 −5 0 5 10
score
Cisplatin
−10 −5 0 5 10
score
Gefitinib
−10 −5 0 5 10
score
Gemcitabine
−10 −5 0 5 10
score
Topotecan
−10 −5 0 5 10
score

Patient 272

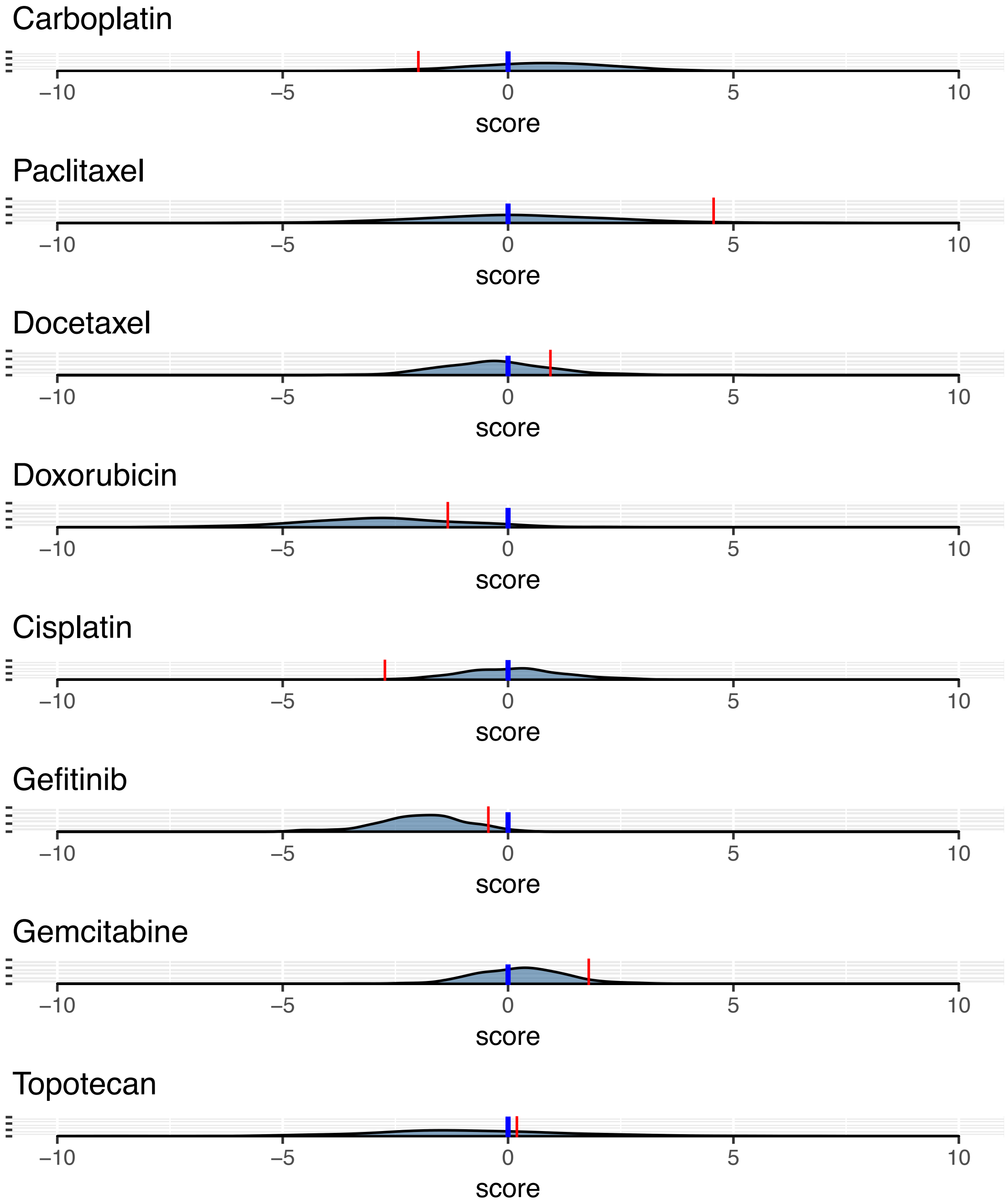

Carboplatin
–10
–5
0
5
10
score
Paclitaxel
–10
–5
0
5
10
score
Docetaxel
–10
–5
0
5
10
score
Doxorubicin
–10
–5
0
5
10
score
Cisplatin
–10
–5
0
5
10
score
Gefitinib
–10
–5
0
5
10
score
Gemcitabine
–10
–5
0
5
10
score
Topotecan
–10
–5
0
5
10
score

Patient 286

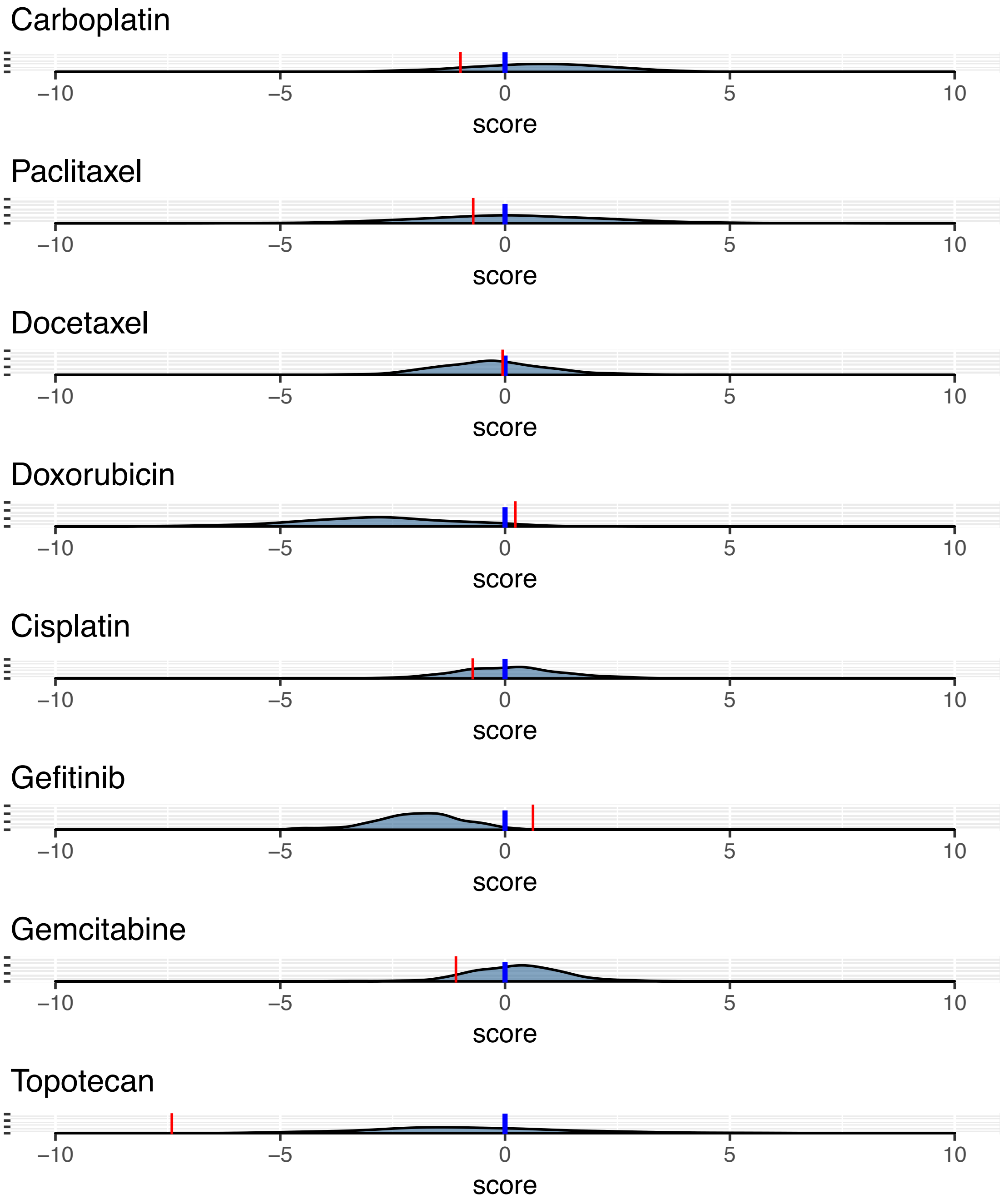
Carboplatin
Paclitaxel
Docetaxel
Doxorubicin
Cisplatin
Gefitinib
Gemcitabine
Topotecan
–10
–5
0
5
10
score

Patient 317

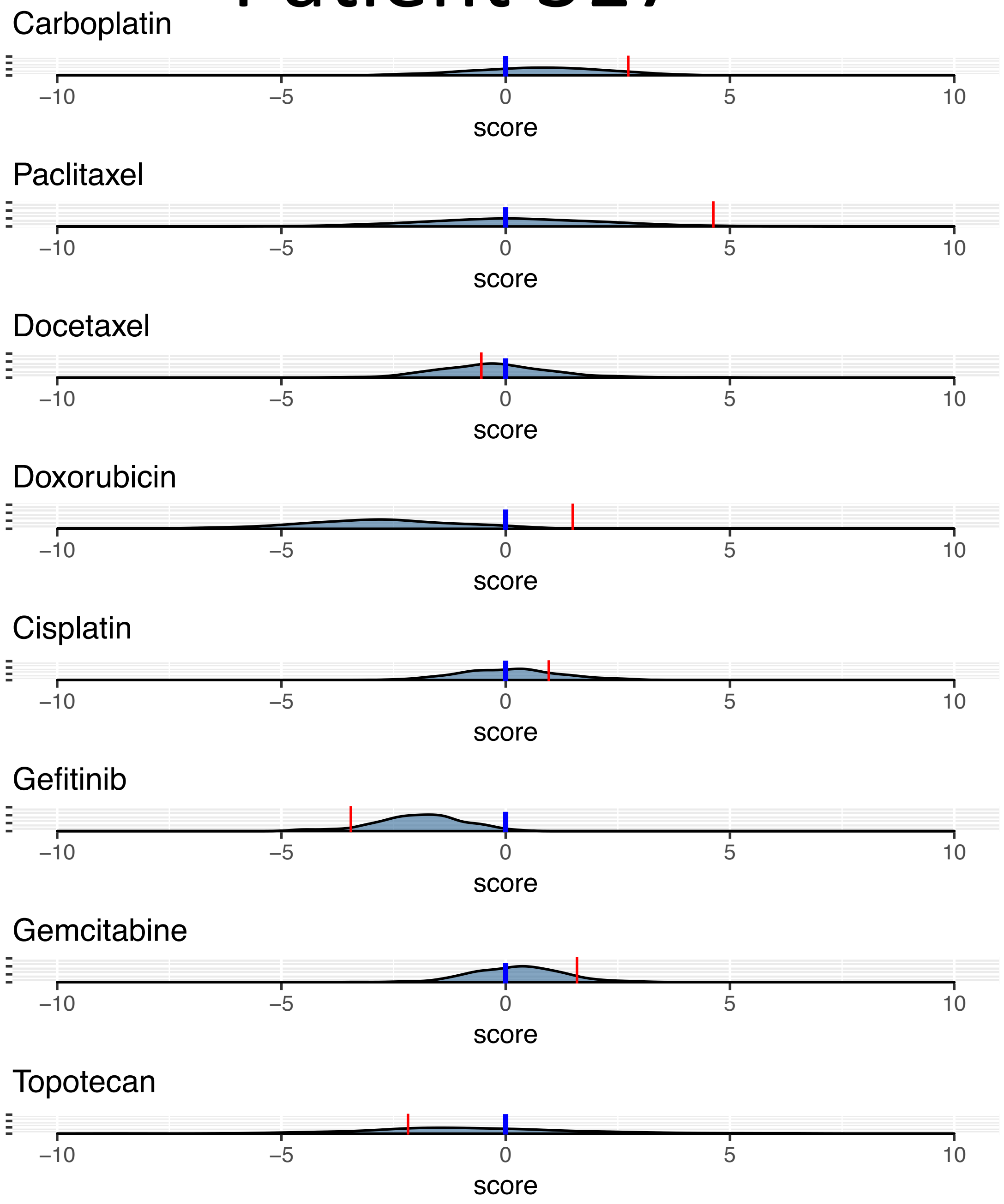

Carboplatin
–10
–5
0
5
10
score
Paclitaxel
–10
–5
0
5
10
score
Docetaxel
–10
–5
0
5
10
score
Doxorubicin
–10
–5
0
5
10
score
Cisplatin
–10
–5
0
5
10
score
Gefitinib
–10
–5
0
5
10
score
Gemcitabine
–10
–5
0
5
10
score
Topotecan
–10
–5
0
5
10
score

Patient 336

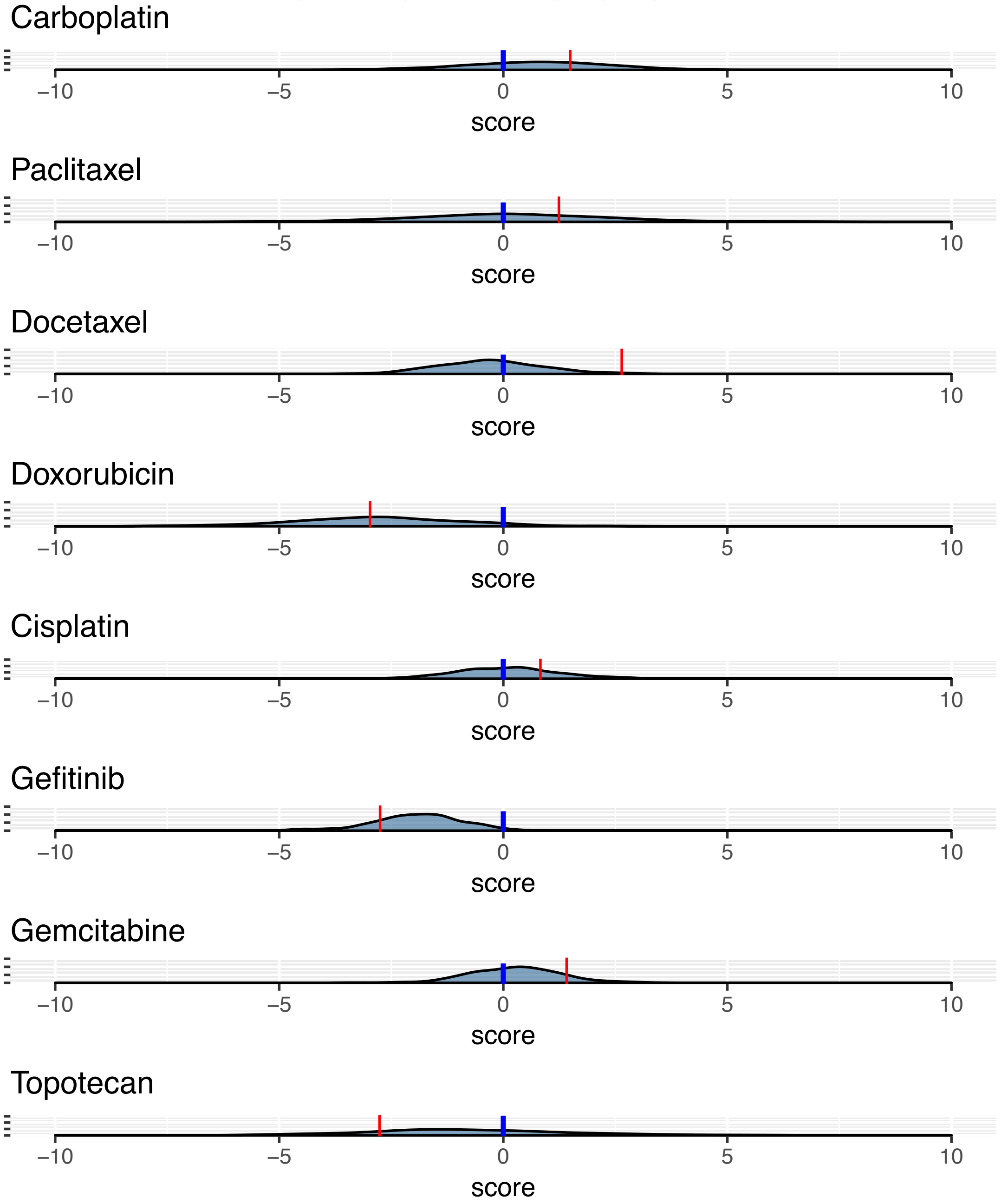

Carboplatin
−10 −5 0 5 10
score
Paclitaxel
−10 −5 0 5 10
score
Docetaxel
−10 −5 0 5 10
score
Doxorubicin
−10 −5 0 5 10
score
Cisplatin
−10 −5 0 5 10
score
Gefitinib
−10 −5 0 5 10
score
Gemcitabine
−10 −5 0 5 10
score
Topotecan
−10 −5 0 5 10
score

Patient 367

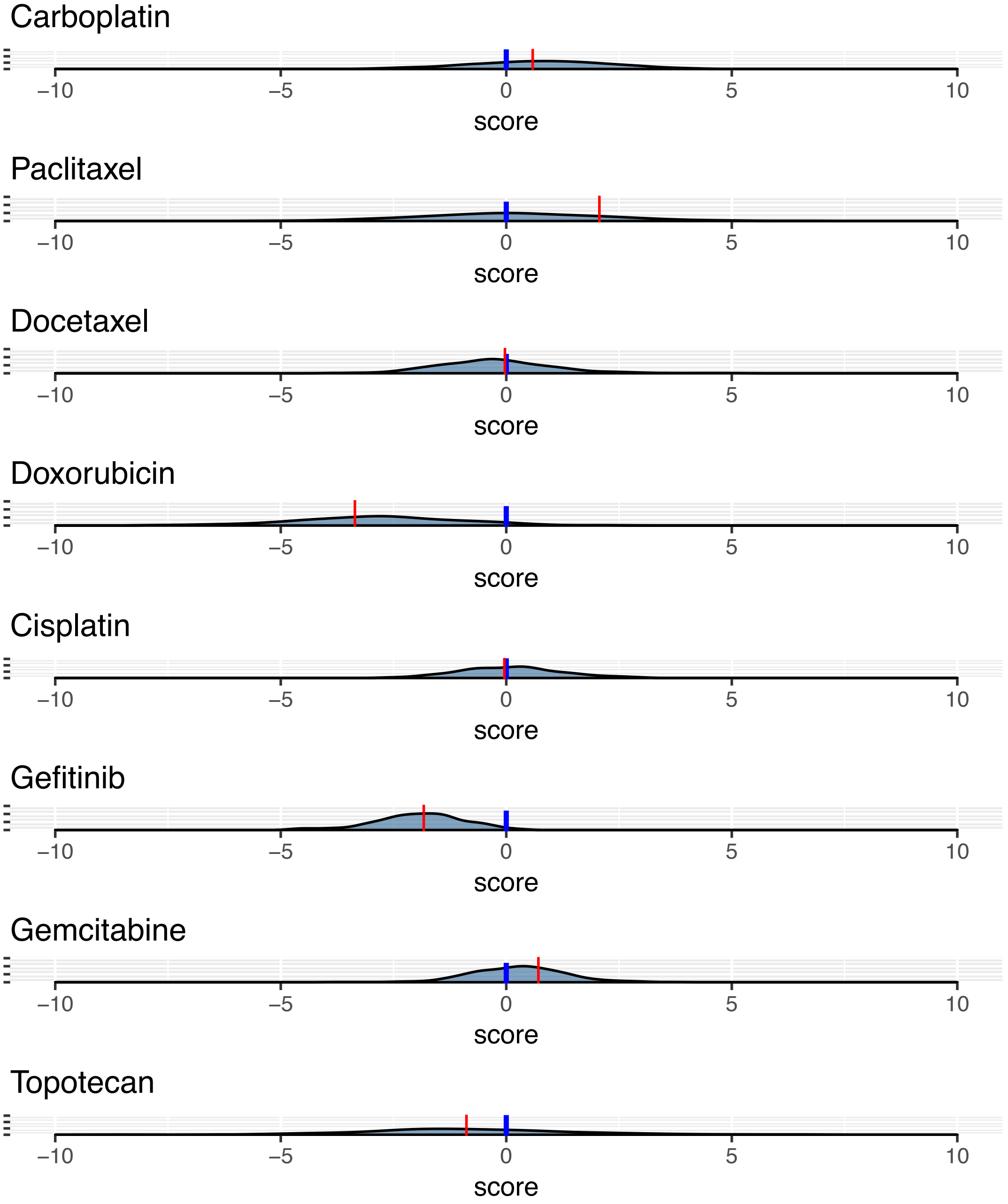

Carboplatin
Paclitaxel
Docetaxel
Doxorubicin
Cisplatin
Gefitinib
Gemcitabine
Topotecan
–10
–5
0
5
10
score

Patient 413

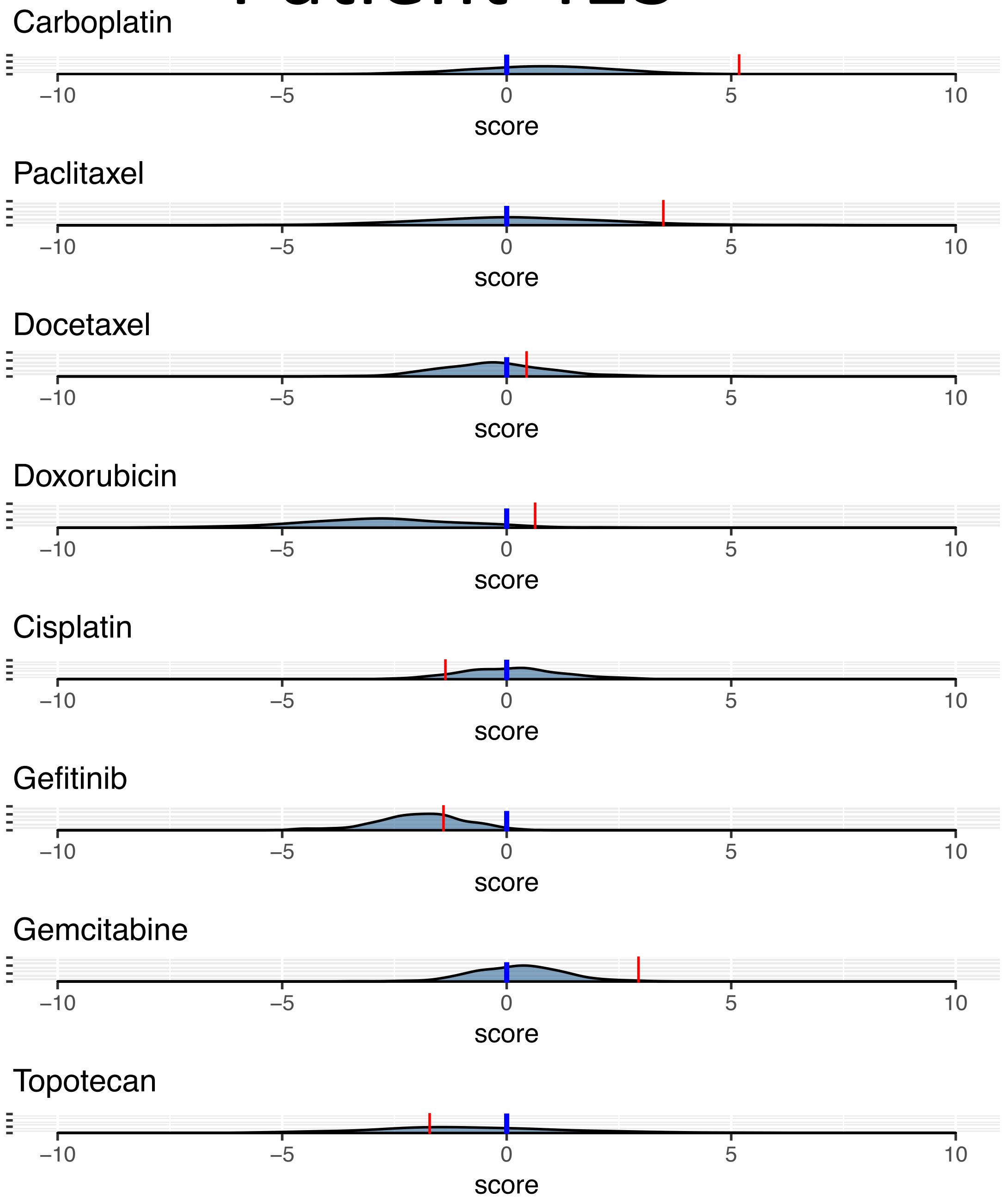

Carboplatin
Paclitaxel
Docetaxel
Doxorubicin
Cisplatin
Gefitinib
Gemcitabine
Topotecan
–10
–5
0
5
10
score

Patient 489

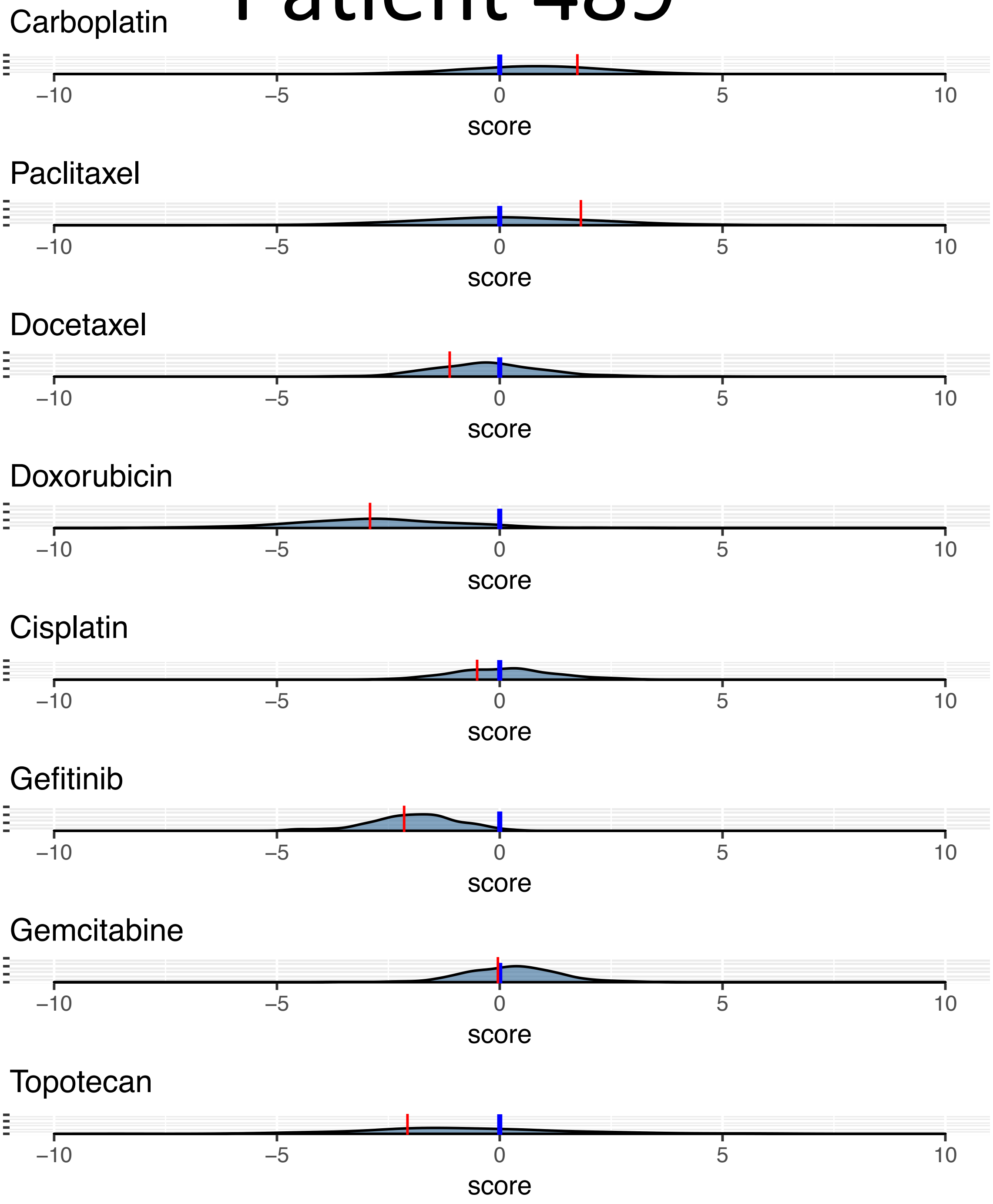

Carboplatin
−10
−5
0
5
10
score
Paclitaxel
score
Docetaxel
score
Doxorubicin
score
Cisplatin
score
Gefitinib
score
Gemcitabine
score
Topotecan
score

Patient 528

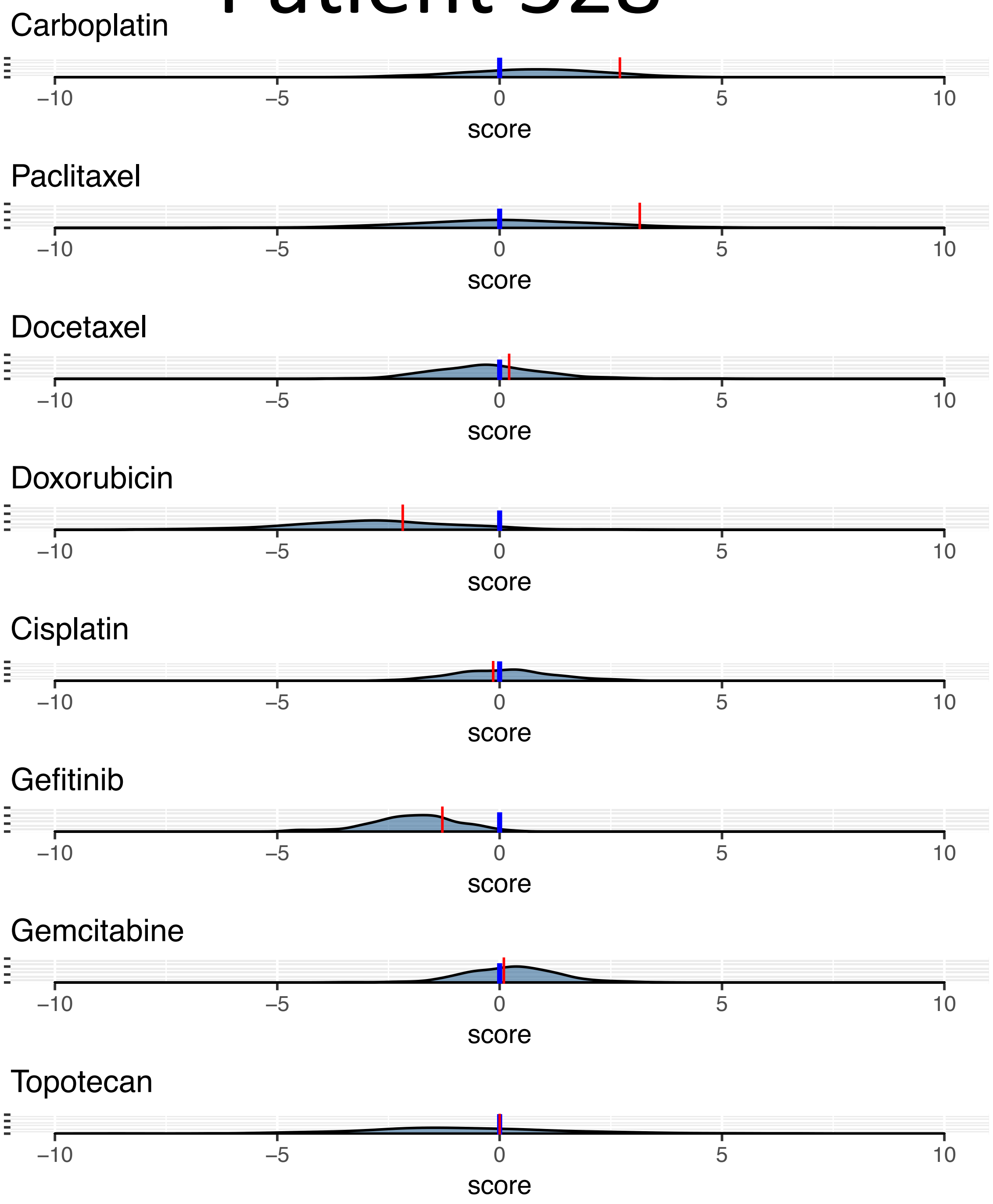
Carboplatin
–10 –5 0 5 10
score
Paclitaxel
–10 –5 0 5 10
score
Docetaxel
–10 –5 0 5 10
score
Doxorubicin
–10 –5 0 5 10
score
Cisplatin
–10 –5 0 5 10
score
Gefitinib
–10 –5 0 5 10
score
Gemcitabine
–10 –5 0 5 10
score
Topotecan
–10 –5 0 5 10
score

Patient 542

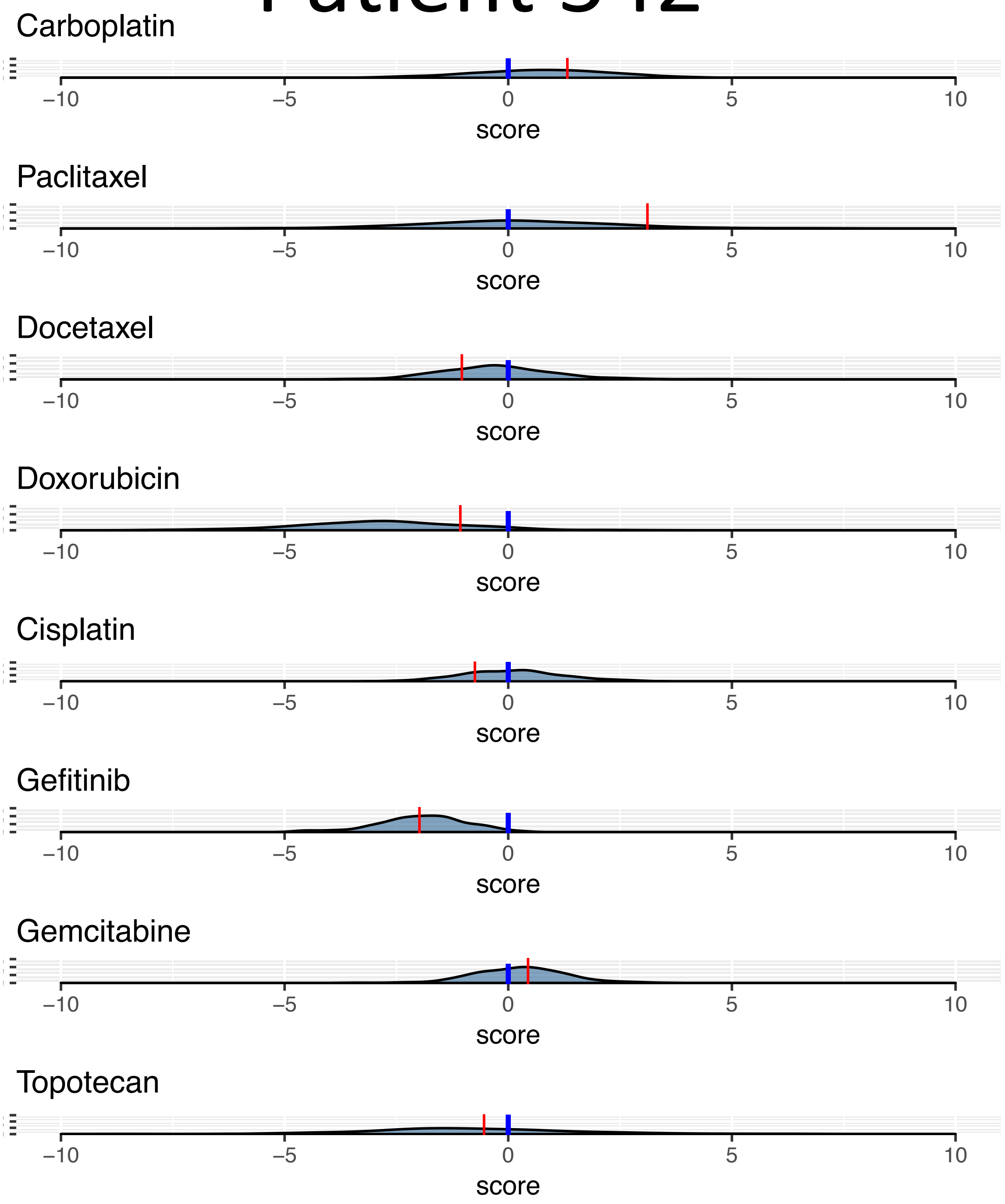
Carboplatin
Paclitaxel
Docetaxel
Doxorubicin
Cisplatin
Gefitinib
Gemcitabine
Topotecan
–10
–5
0
5
10
score

Patient 545

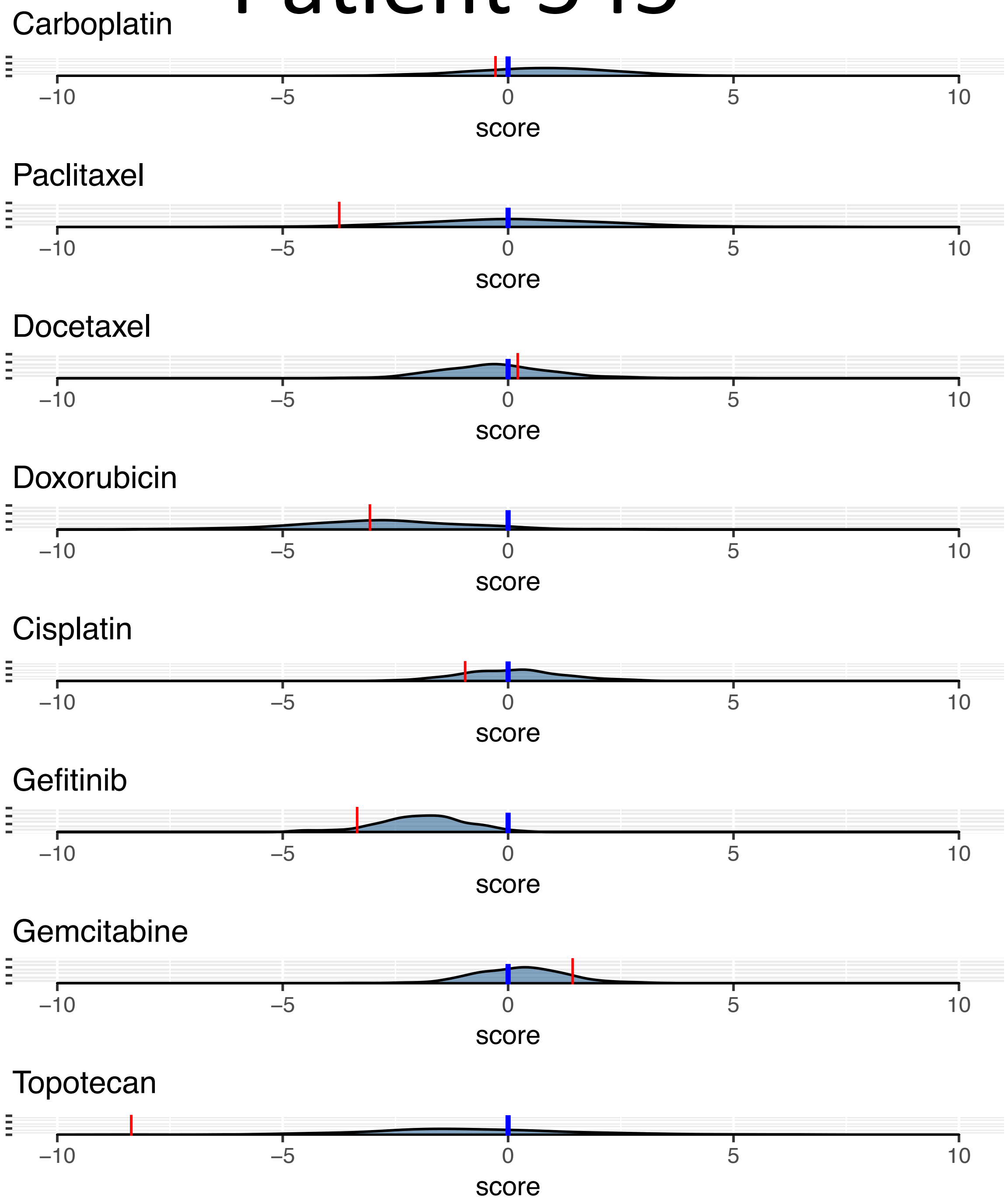

Carboplatin
Paclitaxel
Docetaxel
Doxorubicin
Cisplatin
Gefitinib
Gemcitabine
Topotecan
−10
−5
0
5
10
score

Patient 588

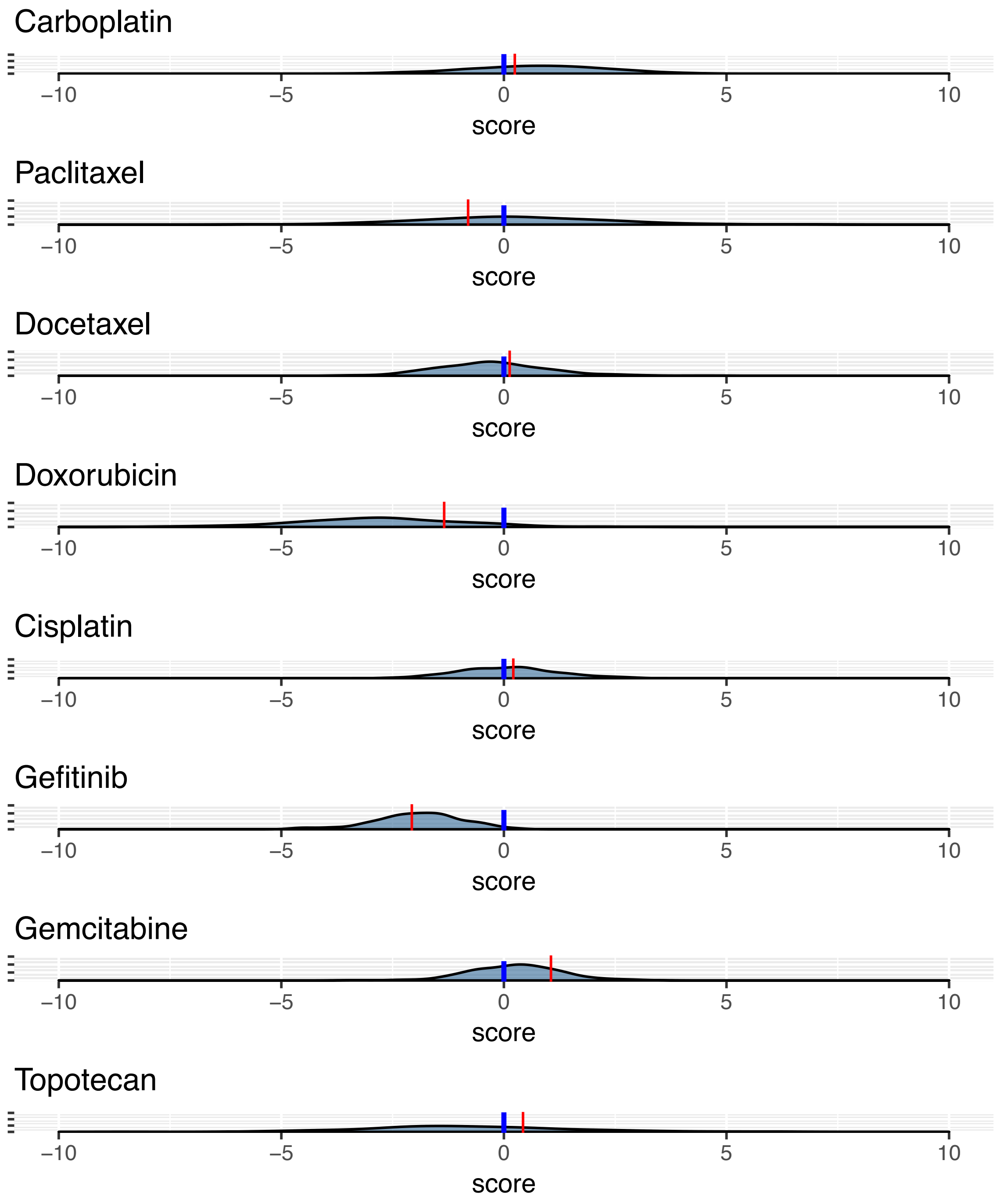

Carboplatin
−10
−5
0
5
10
score
Paclitaxel
−10
−5
0
5
10
score
Docetaxel
−10
−5
0
5
10
score
Doxorubicin
−10
−5
0
5
10
score
Cisplatin
−10
−5
0
5
10
score
Gefitinib
−10
−5
0
5
10
score
Gemcitabine
−10
−5
0
5
10
score
Topotecan
−10
−5
0
5
10
score

Patient 617

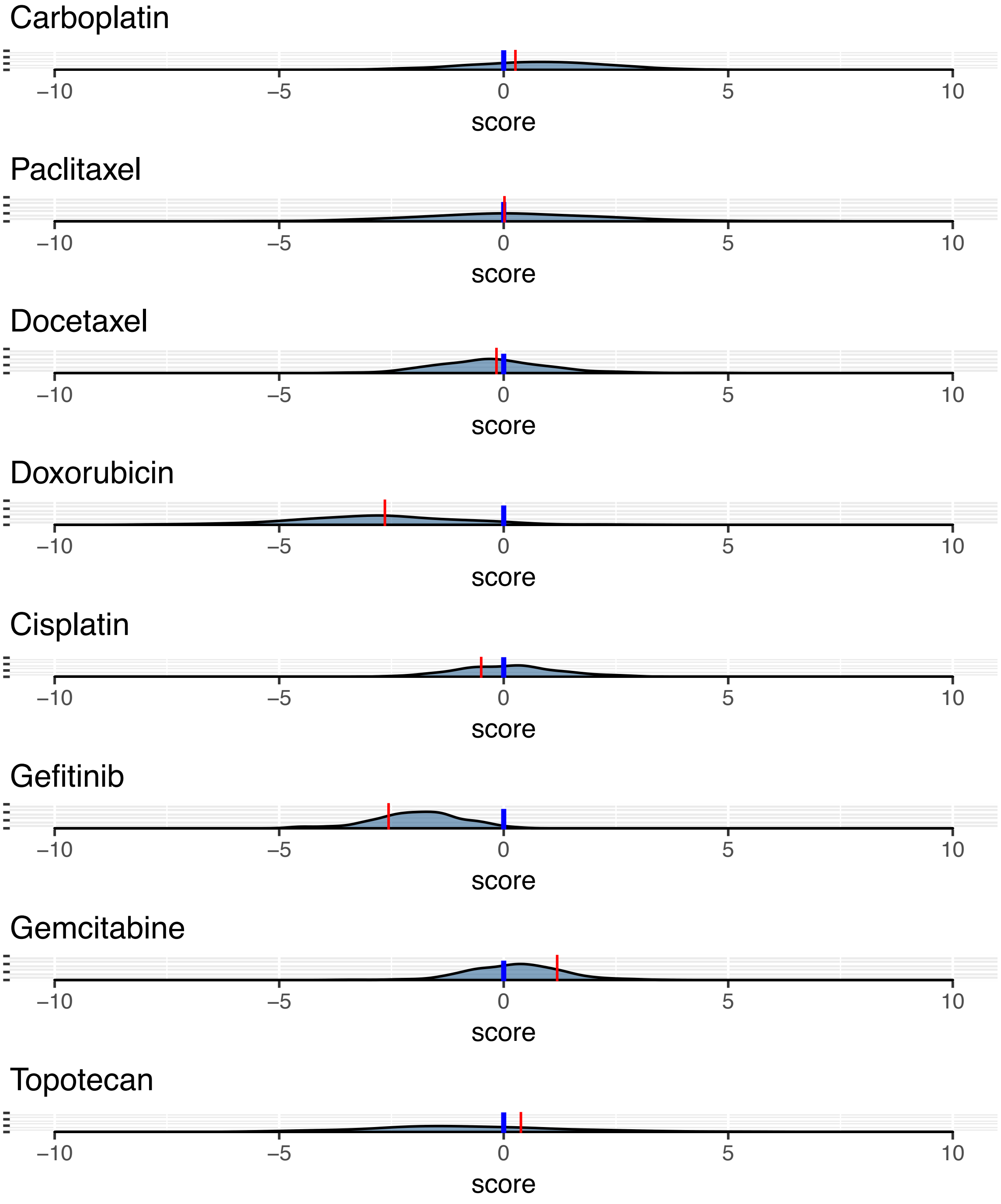

Carboplatin
–10 –5 0 5 10
score
Paclitaxel
–10 –5 0 5 10
score
Docetaxel
–10 –5 0 5 10
score
Doxorubicin
–10 –5 0 5 10
score
Cisplatin
–10 –5 0 5 10
score
Gefitinib
–10 –5 0 5 10
score
Gemcitabine
–10 –5 0 5 10
score
Topotecan
–10 –5 0 5 10
score

Patient 620

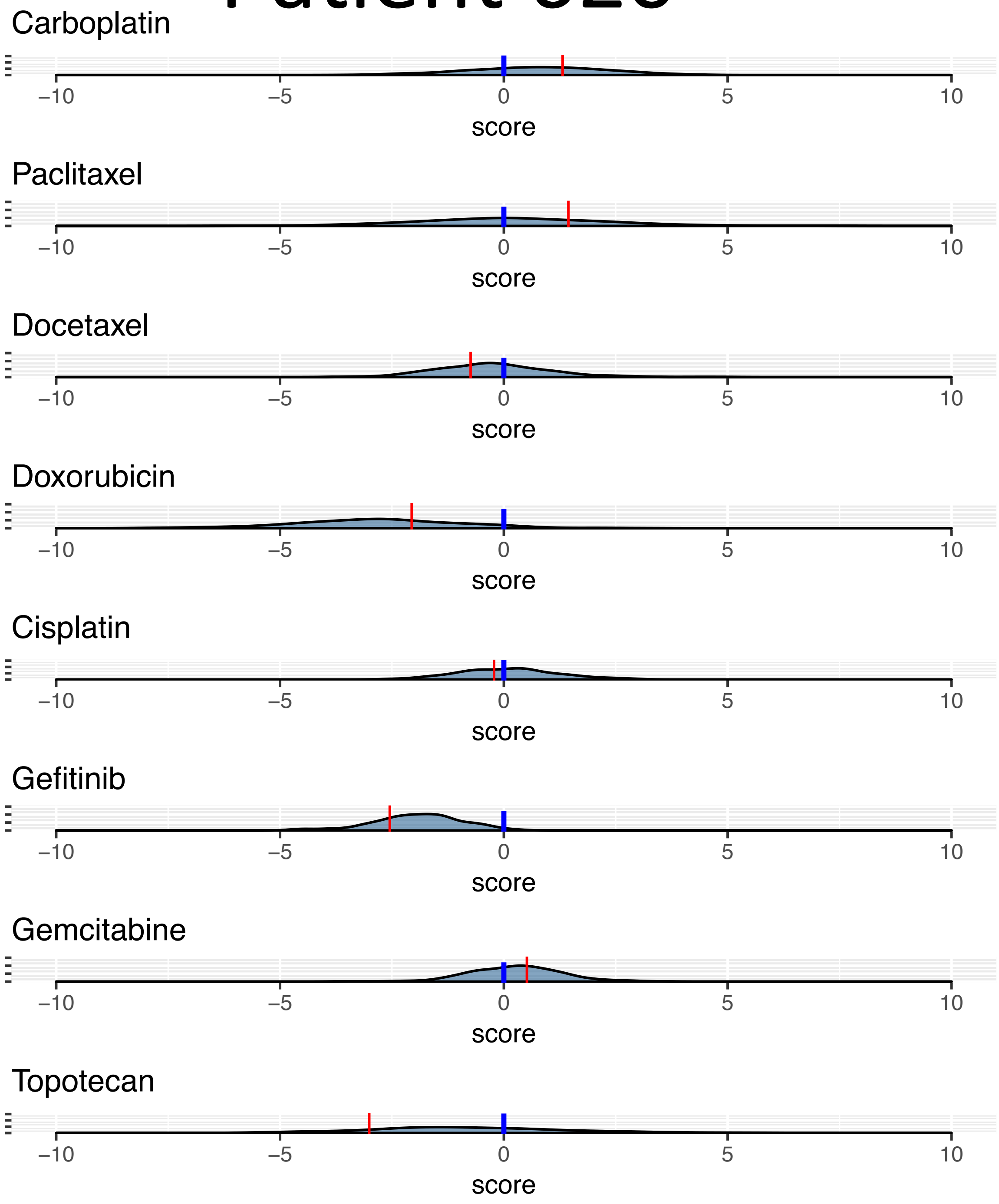
Carboplatin
Paclitaxel
Docetaxel
Doxorubicin
Cisplatin
Gefitinib
Gemcitabine
Topotecan
–10
–5
0
5
10
score

Patient 813

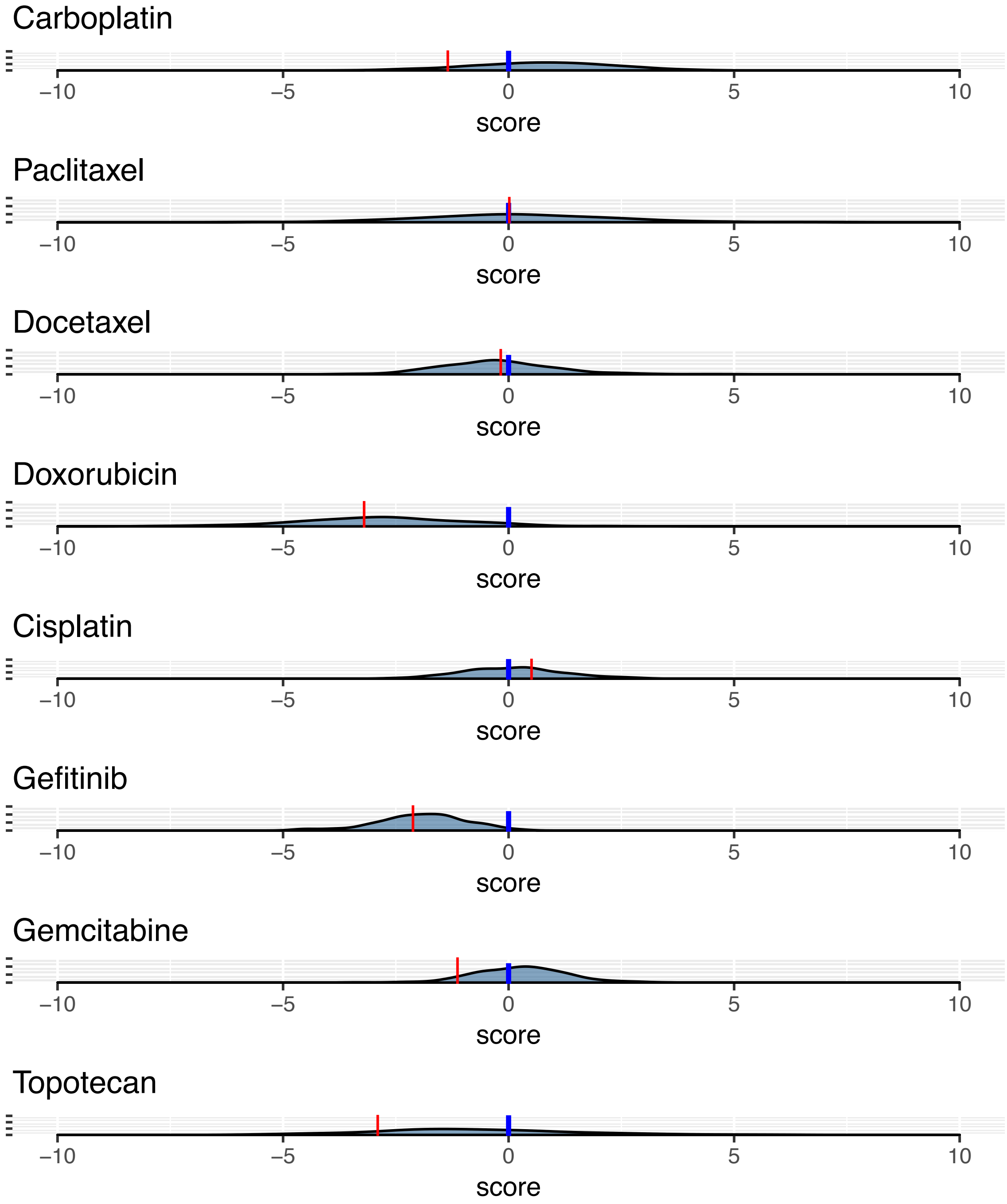

Carboplatin
Paclitaxel
Docetaxel
Doxorubicin
Cisplatin
Gefitinib
Gemcitabine
Topotecan
−10
−5
0
5
10
score

Patient 992

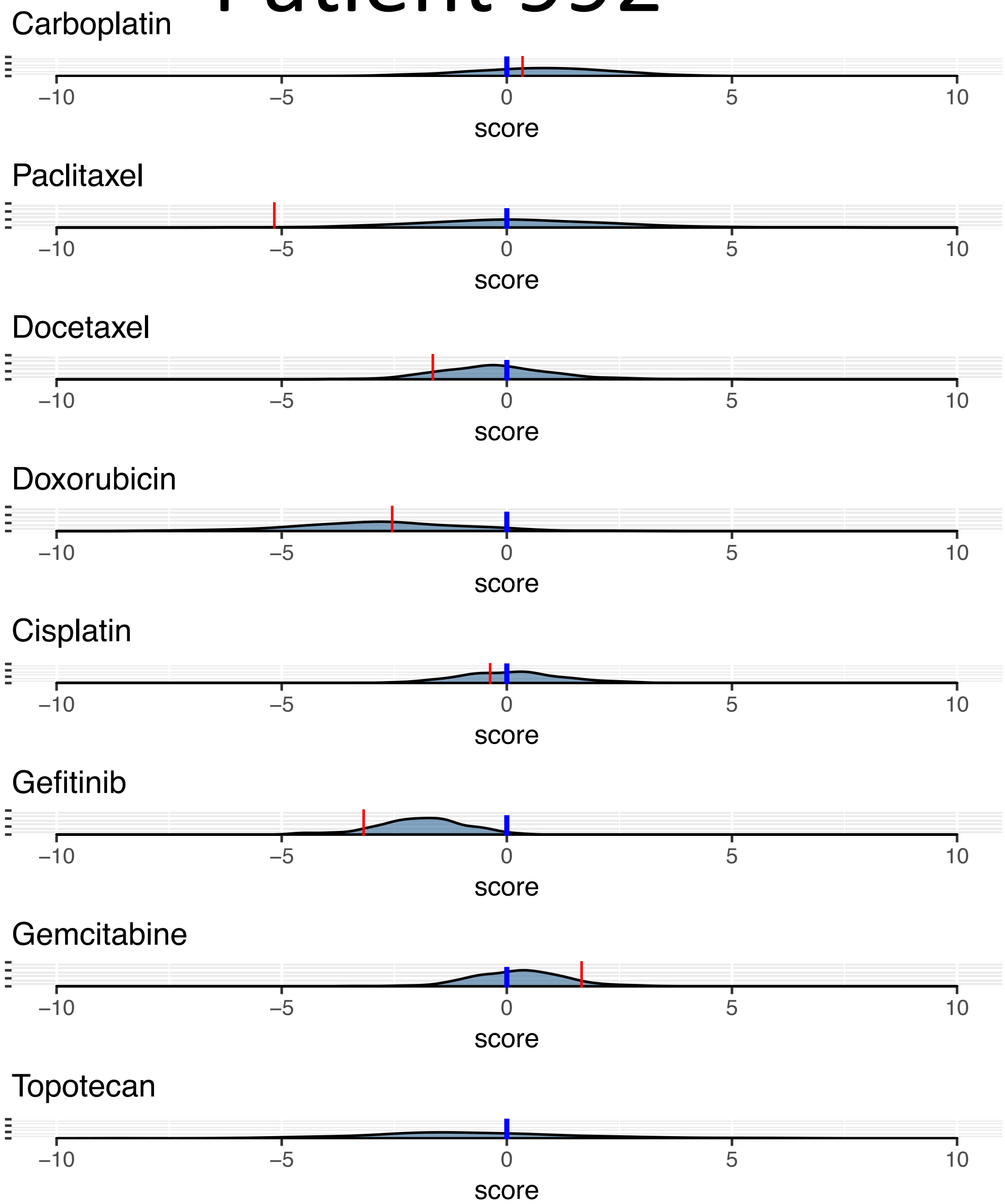

Carboplatin
–10
–5
0
5
10
score
Paclitaxel
–10
–5
0
5
10
score
Docetaxel
–10
–5
0
5
10
score
Doxorubicin
–10
–5
0
5
10
score
Cisplatin
–10
–5
0
5
10
score
Gefitinib
–10
–5
0
5
10
score
Gemcitabine
–10
–5
0
5
10
score
Topotecan
–10
–5
0
5
10
score

Patient 1012

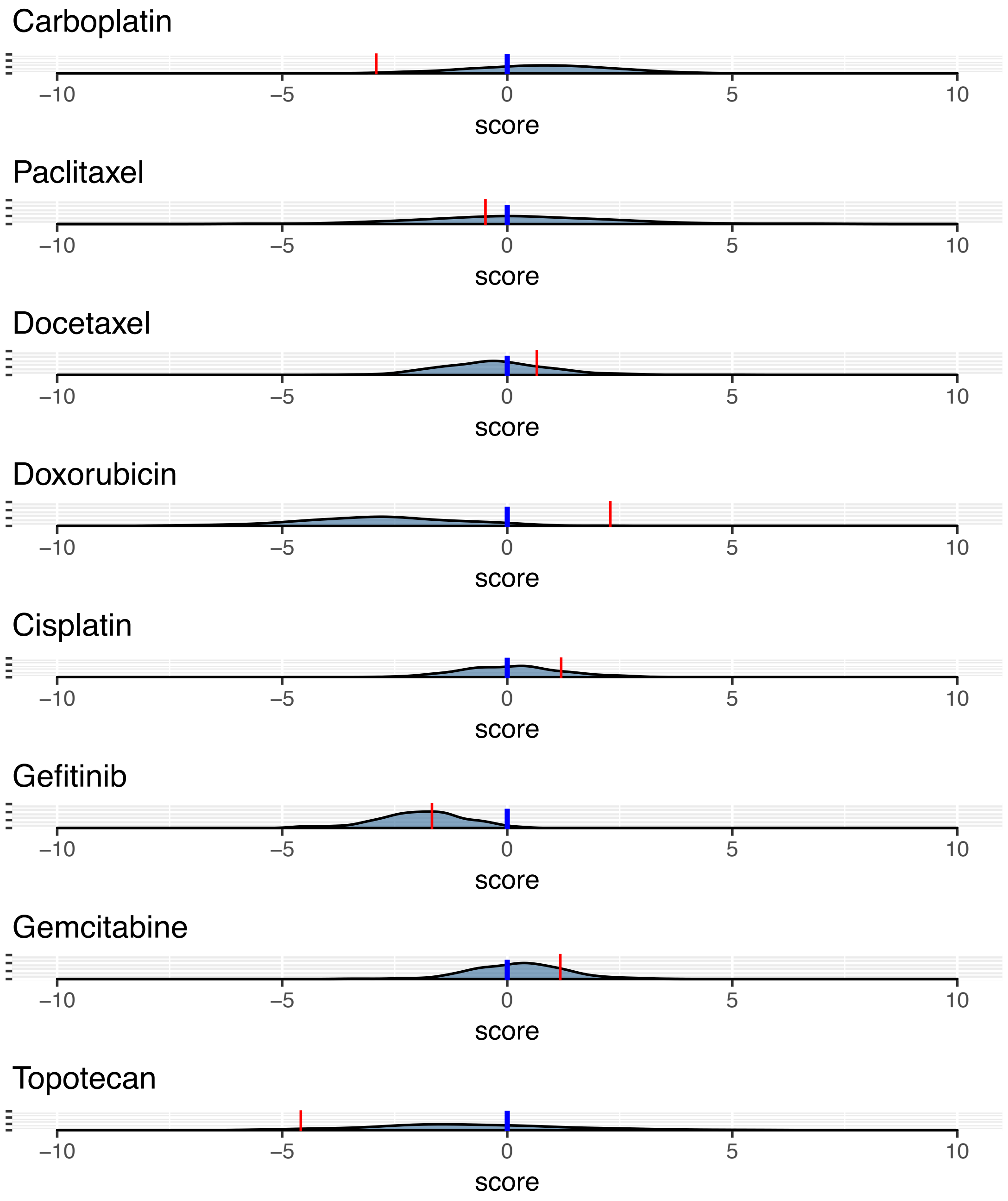

Patient 1122

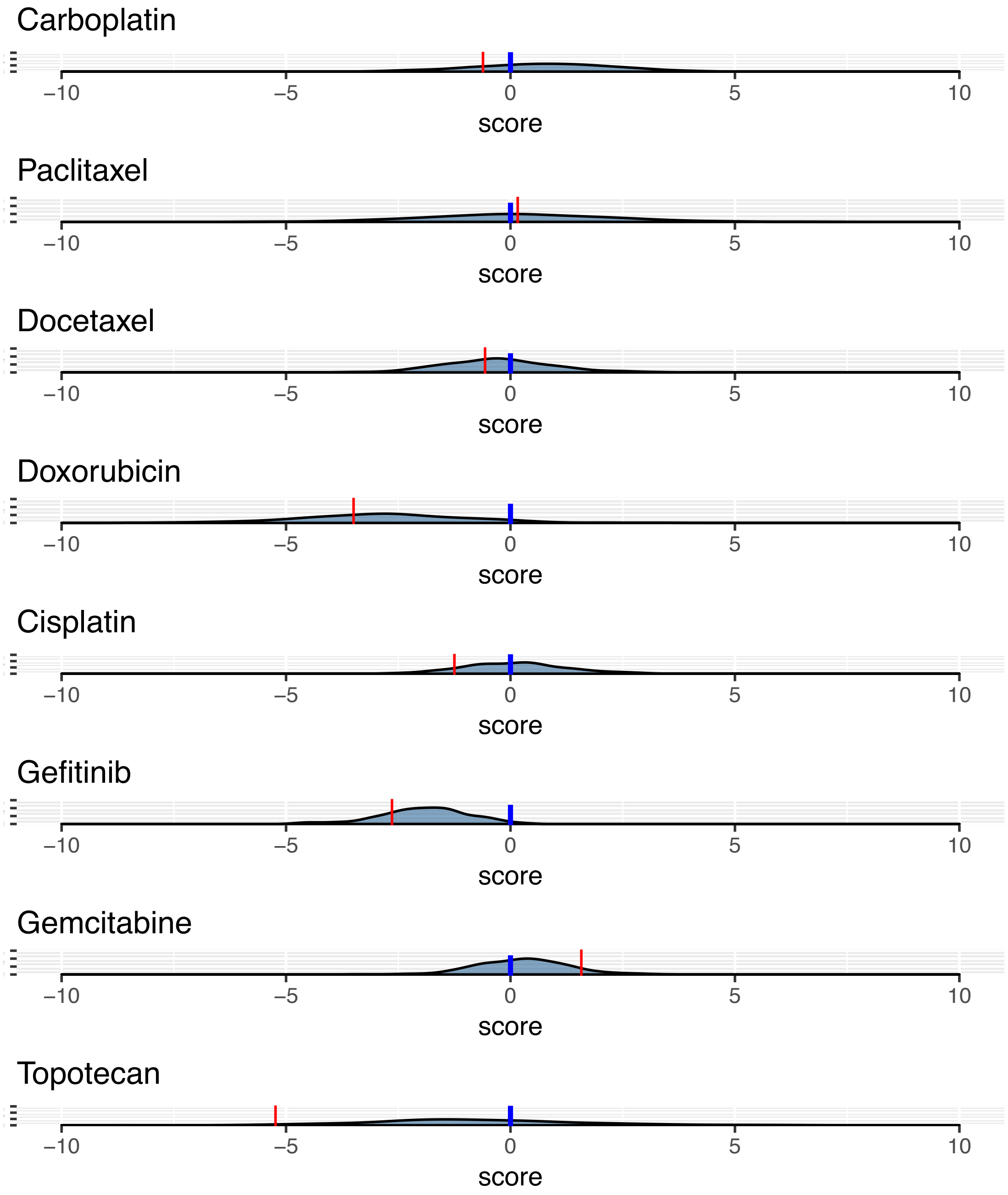

Patient 1129

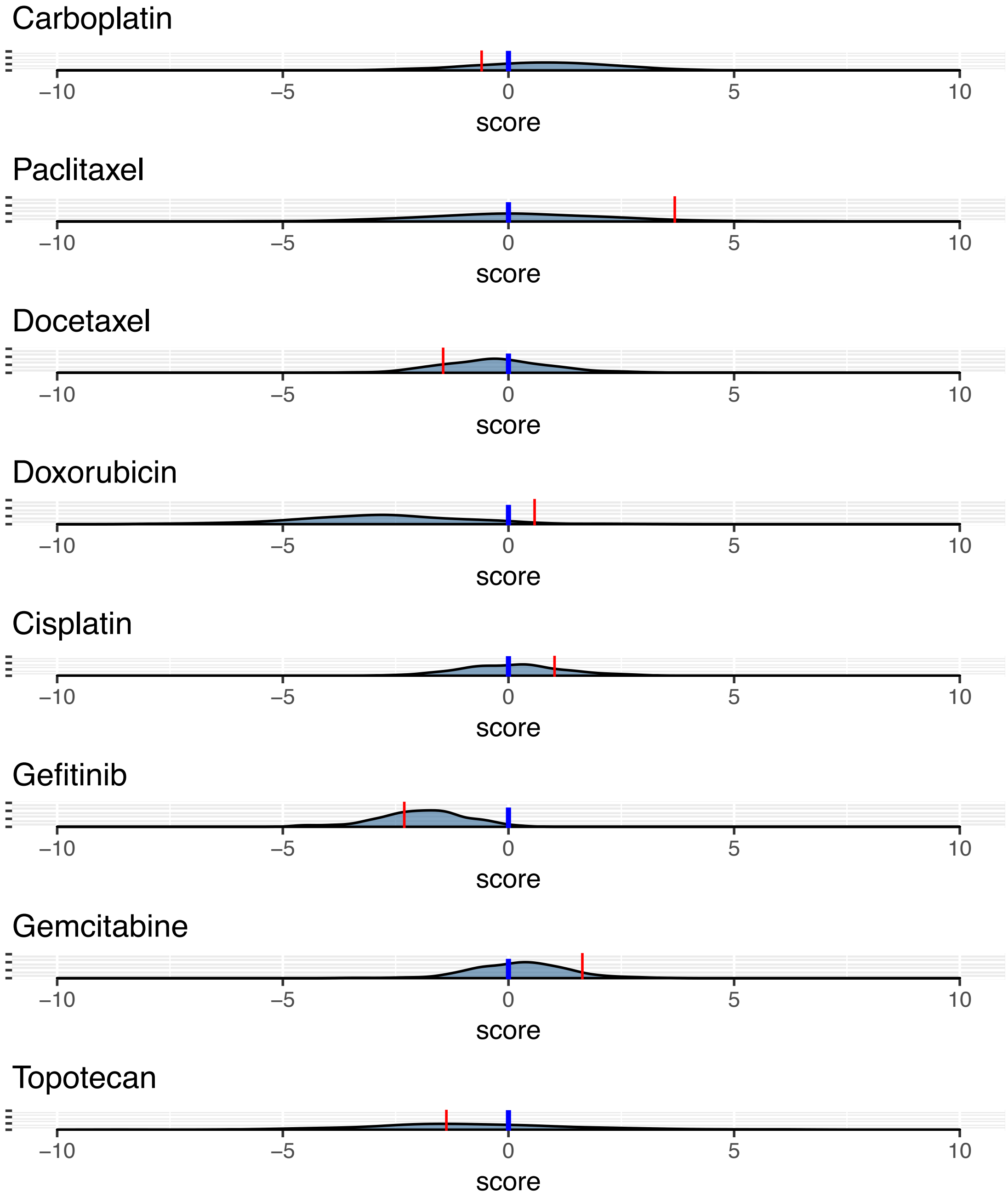

Patient 1145

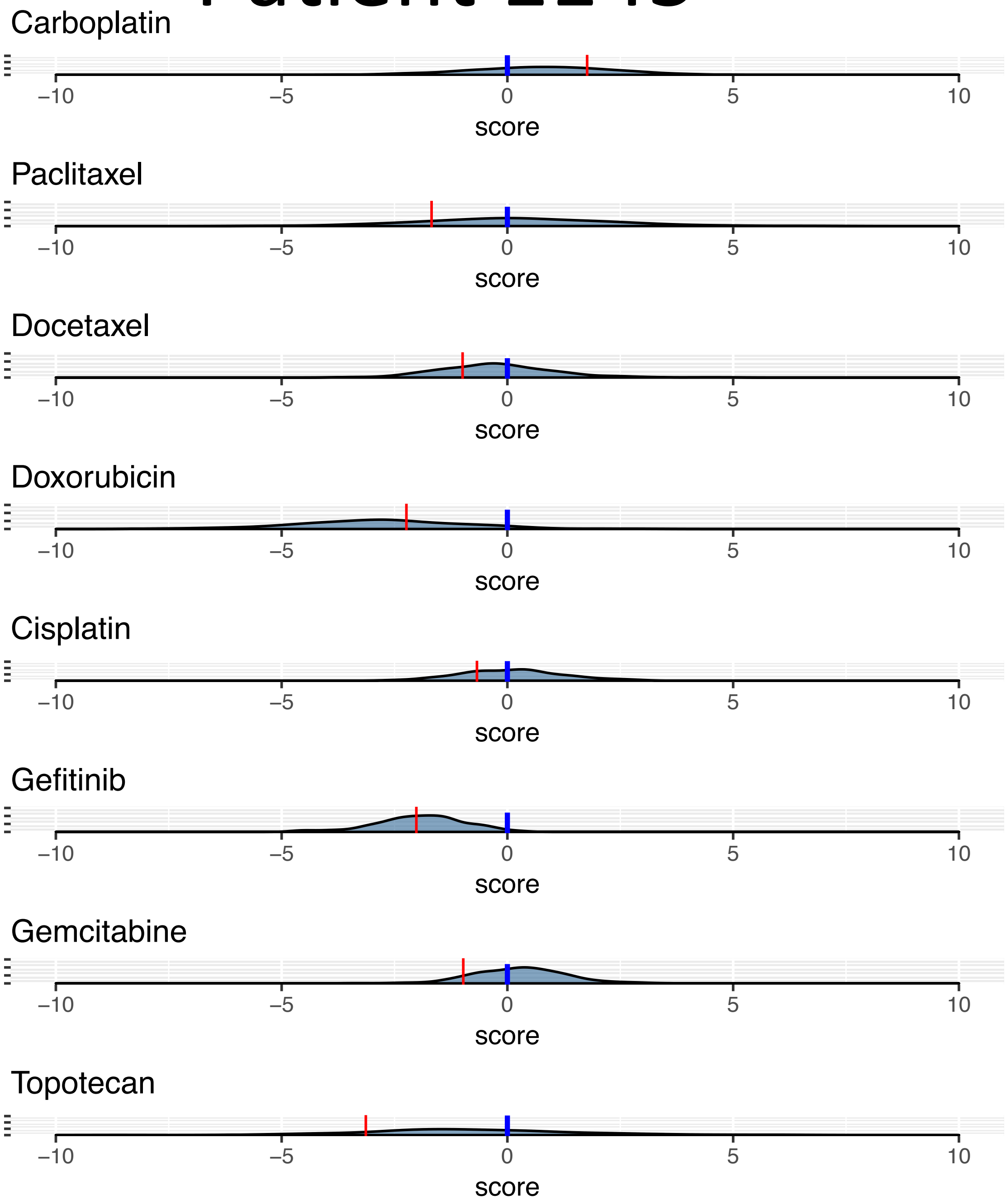

Patient BJ1

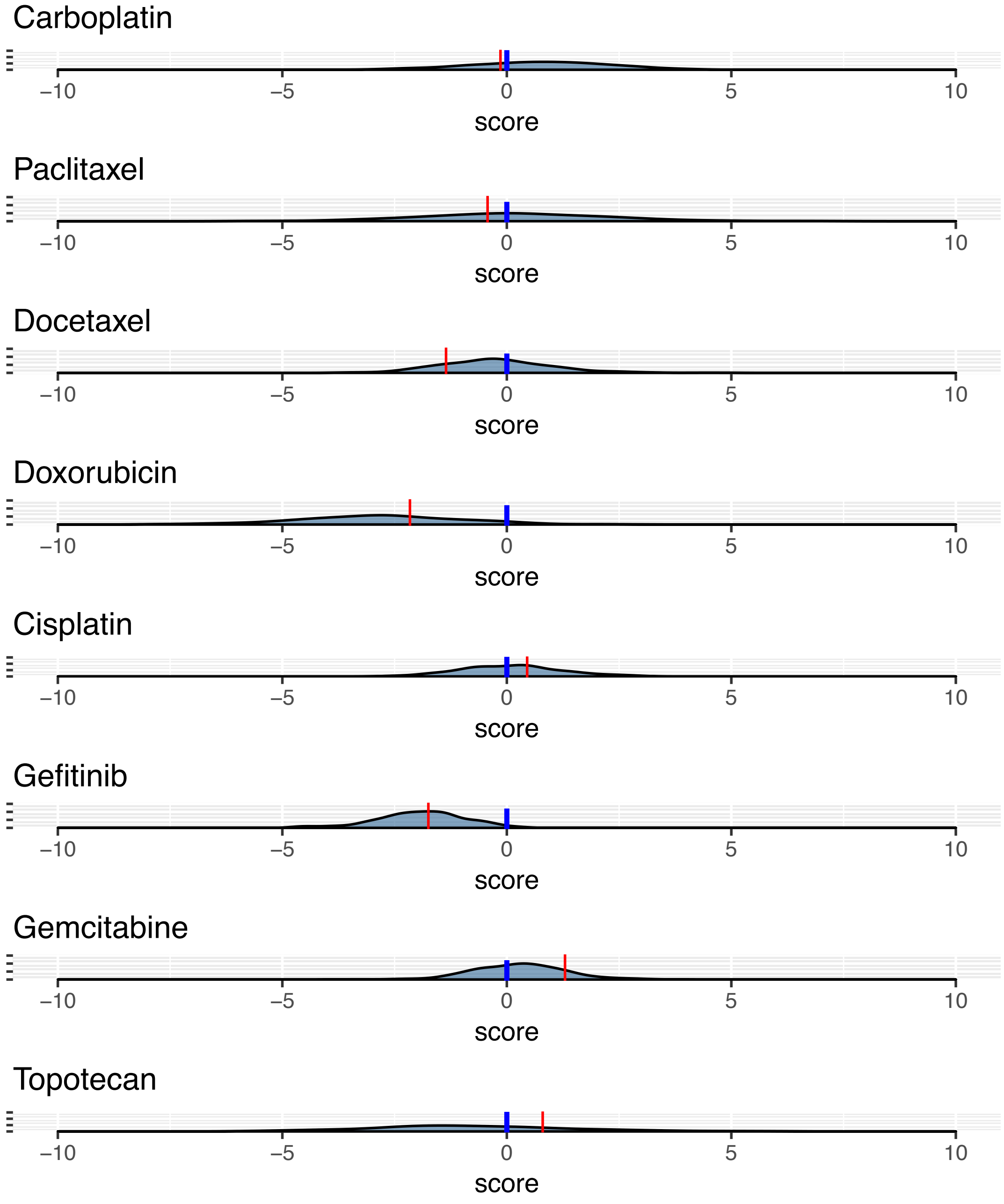

Carboplatin
Paclitaxel
Docetaxel
Doxorubicin
Cisplatin
Gefitinib
Gemcitabine
Topotecan
−10
−5
0
5
10
score

Patient BJ4

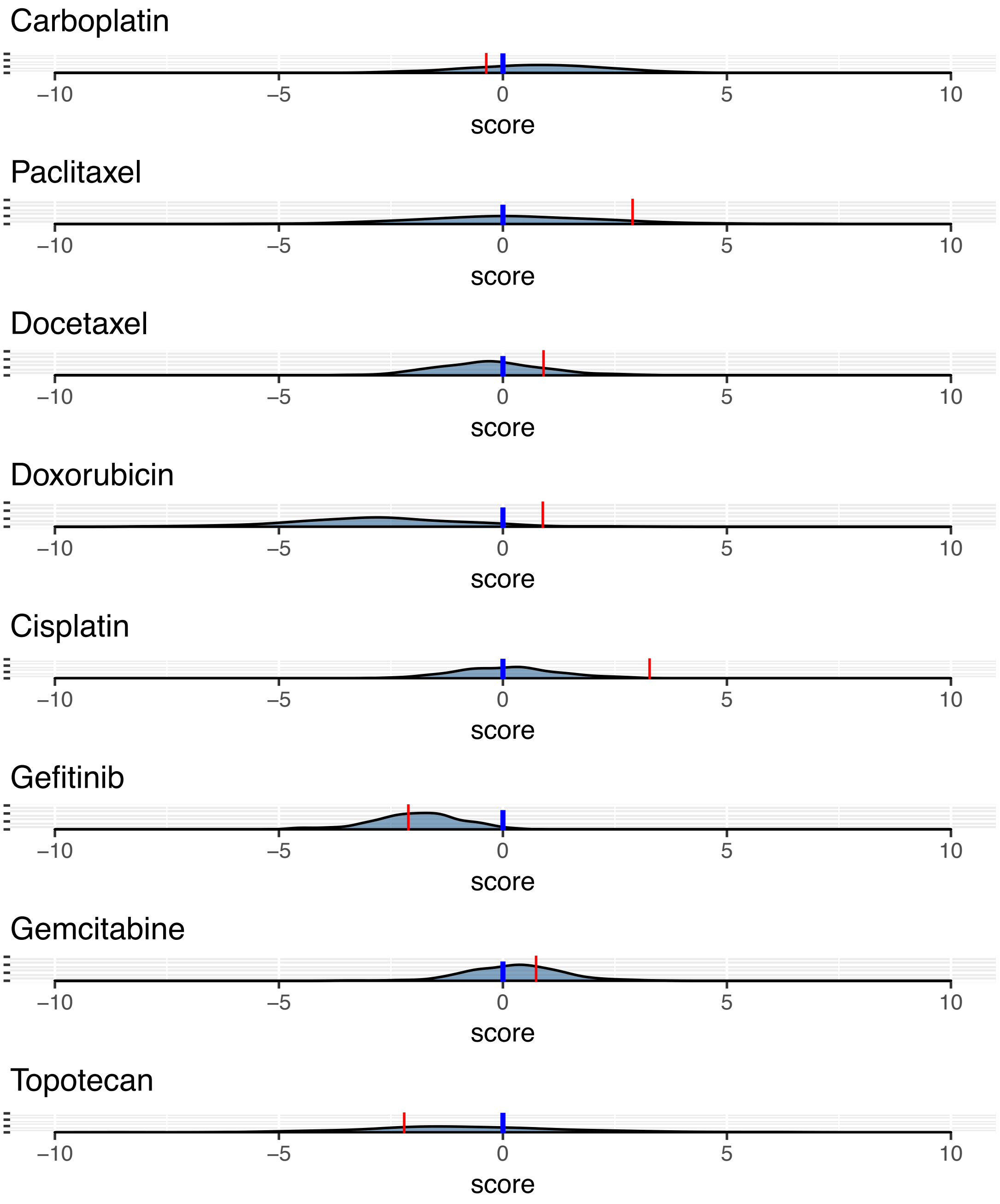
Carboplatin
Paclitaxel
Docetaxel
Doxorubicin
Cisplatin
Gefitinib
Gemcitabine
Topotecan
–10
–5
0
5
10
score

Supplementary Fig. S2. Patients are considered to be responsive to treatments if their respective CA-125 values dropped below normal values (<35; dashed blue line; dashed red line = day of surgery).

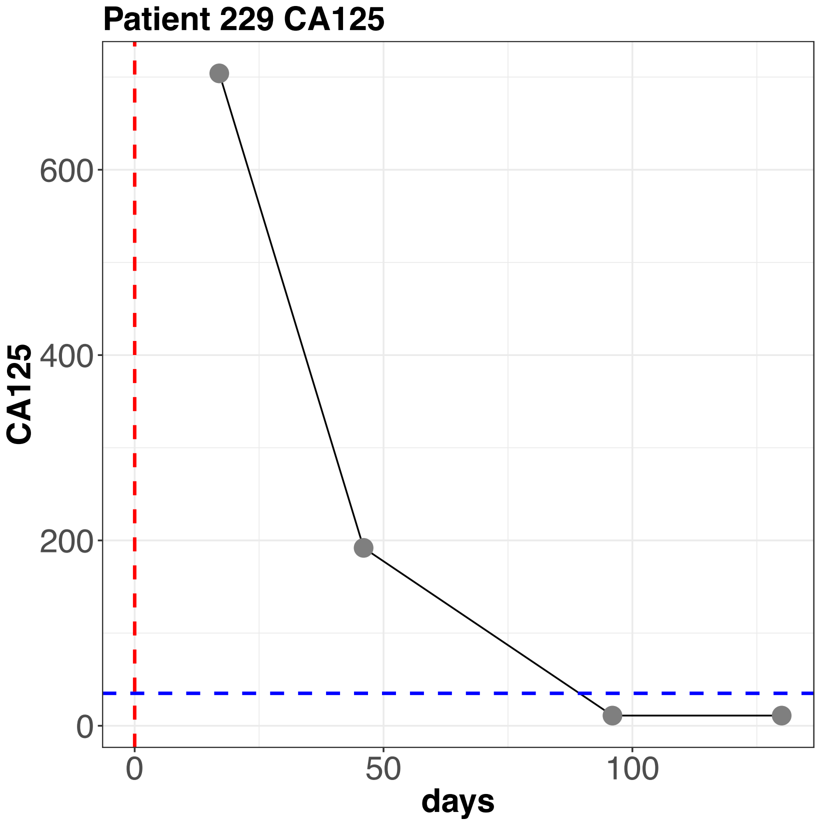

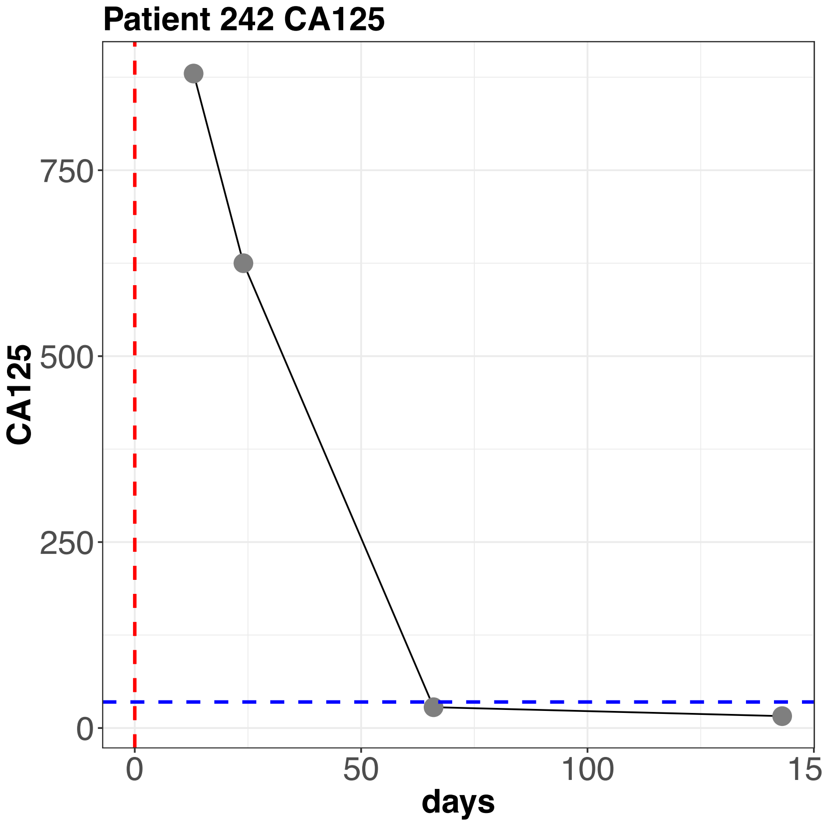

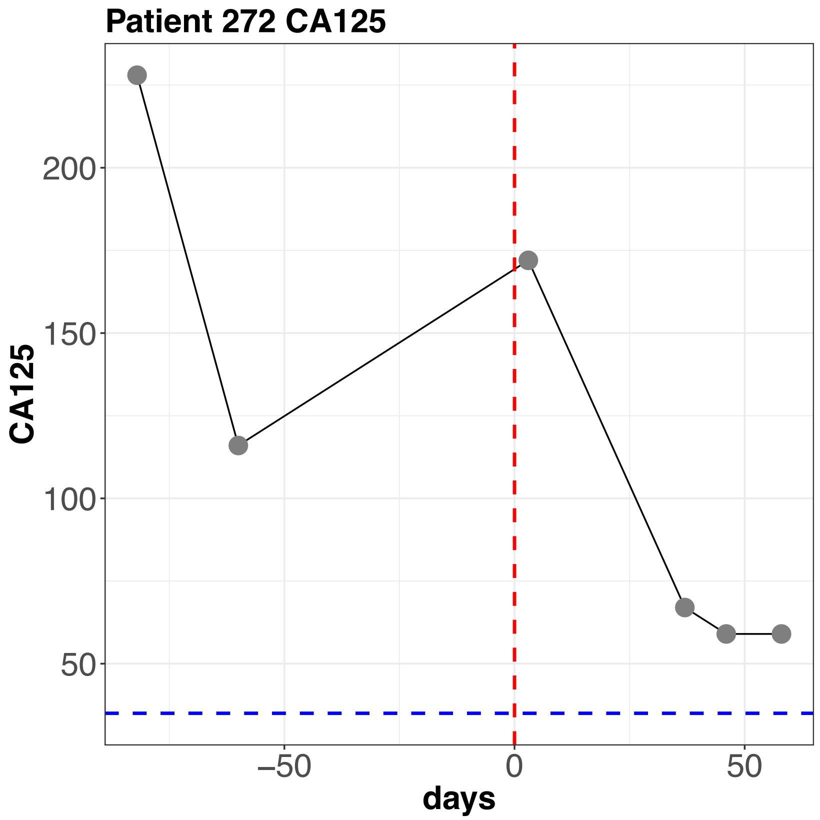
Patient 272 CA125
CA125
200
150
100
50
−50
0
50
days

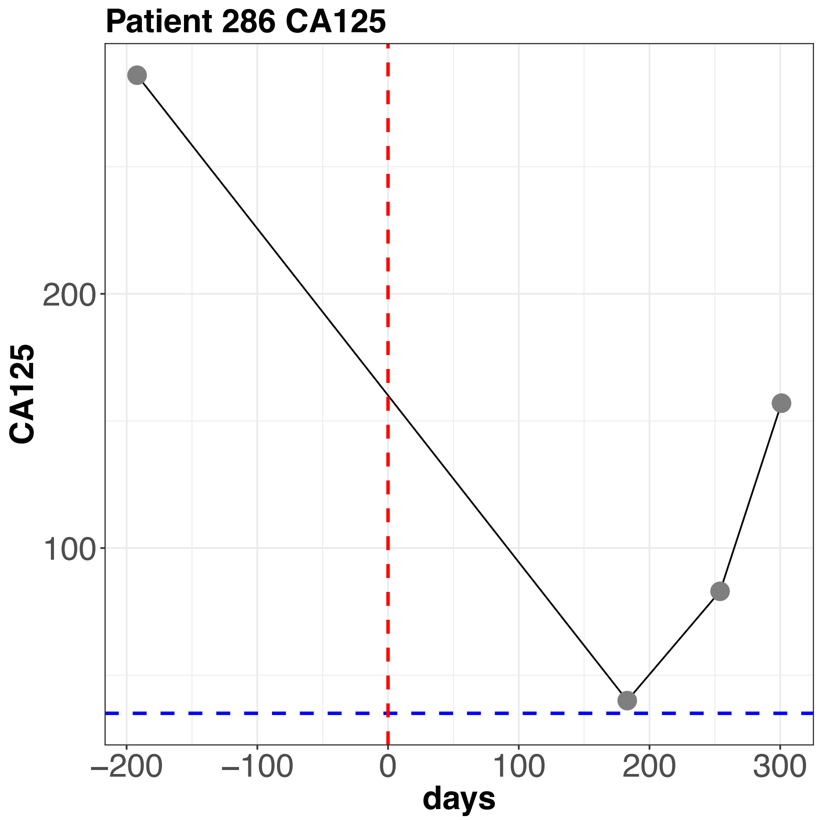
Patient 286 CA125
CA125
200
100
−200
−100
0
100
200
300
days

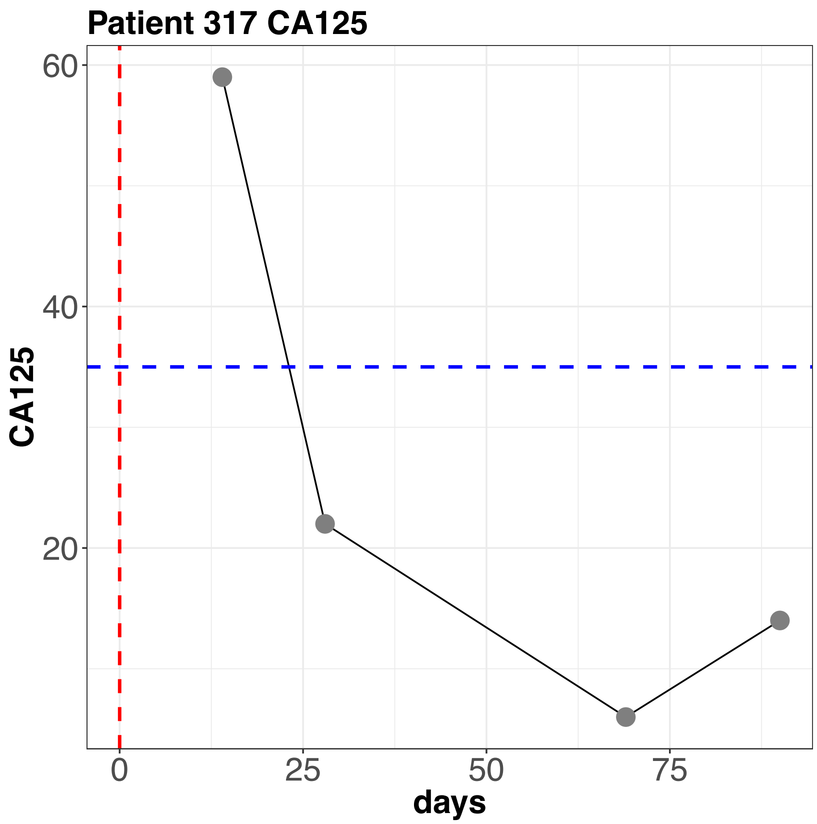
Patient 317 CA125
60
40
20
CA125
0
25
50
75
days

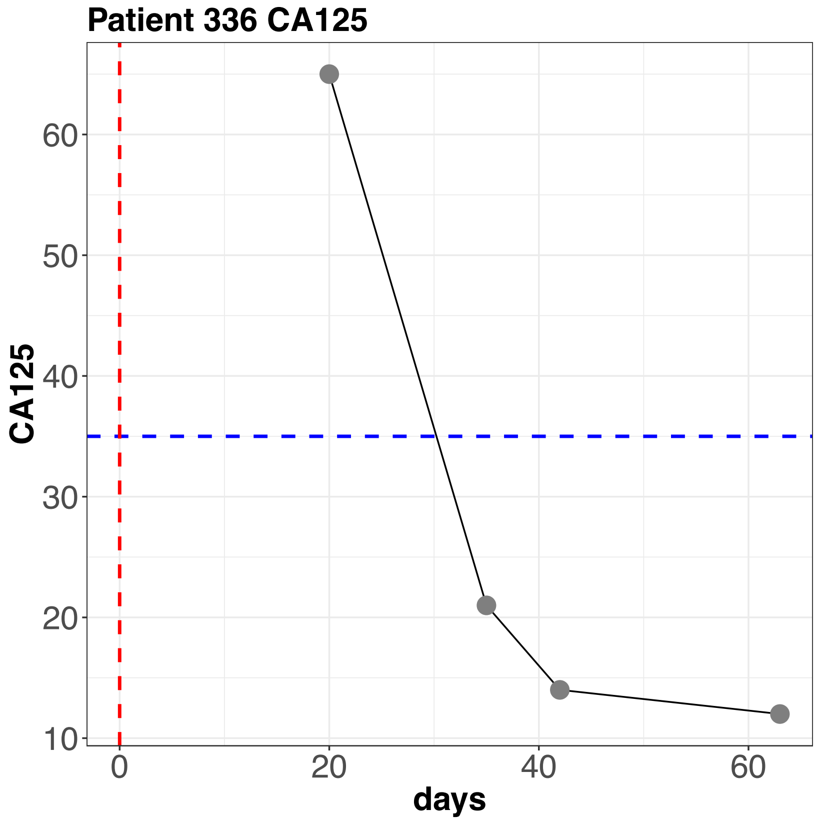
Patient 336 CA125
60
50
40
30
20
10
CA125
0
20
40
60
days

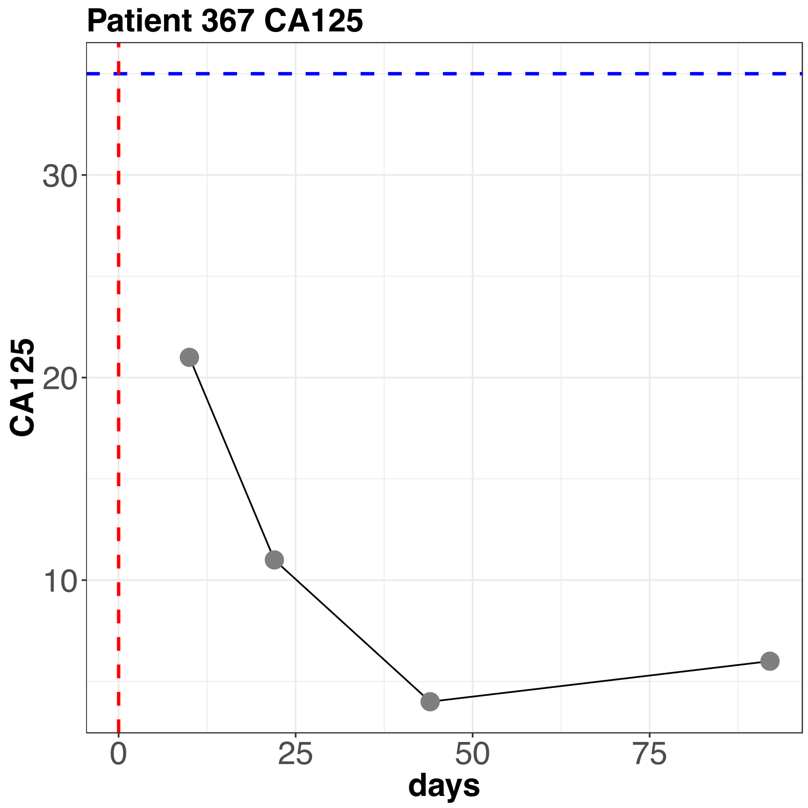

Patient 367 CA125
CA125
30
20
10
0
25
50
75
days

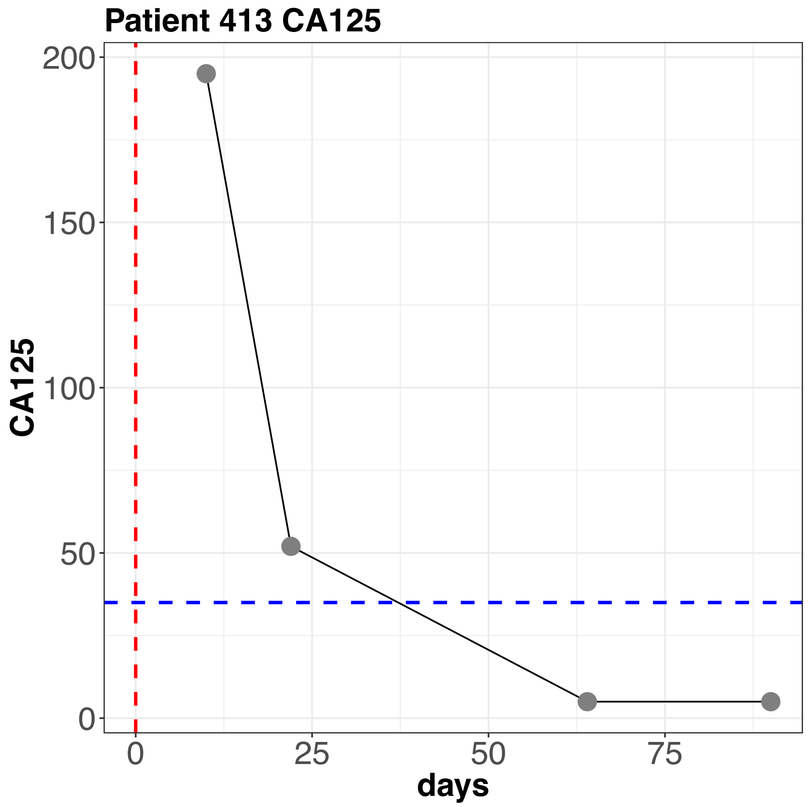

Patient 413 CA125
CA125
200
150
100
50
0
0
25
50
75
days

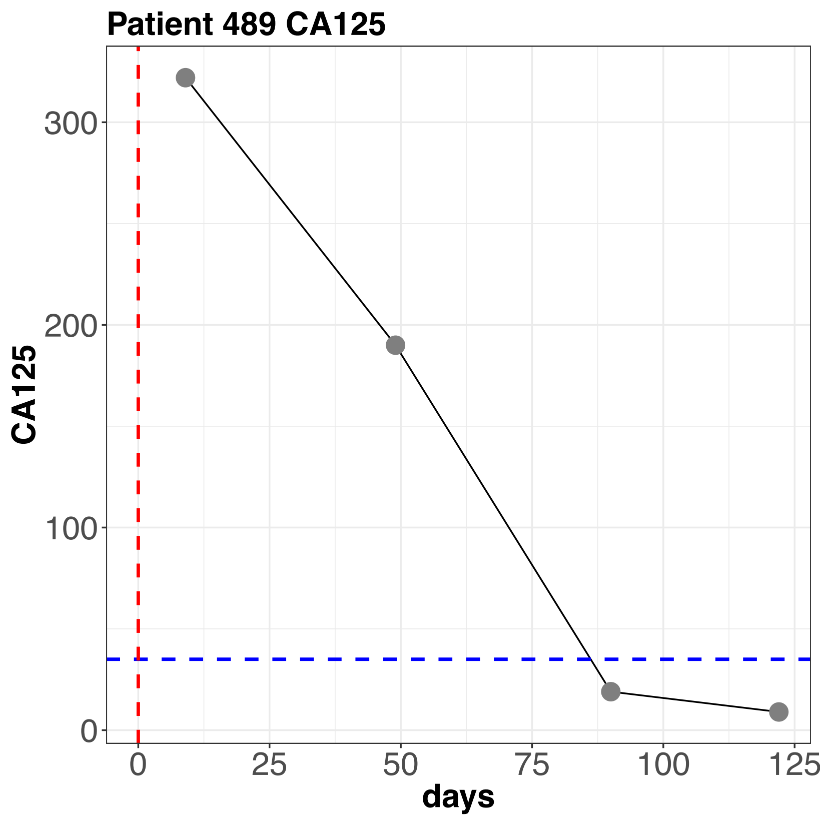
Patient 489 CA125
CA125
300
200
100
0
0
25
50
75
100
125
days

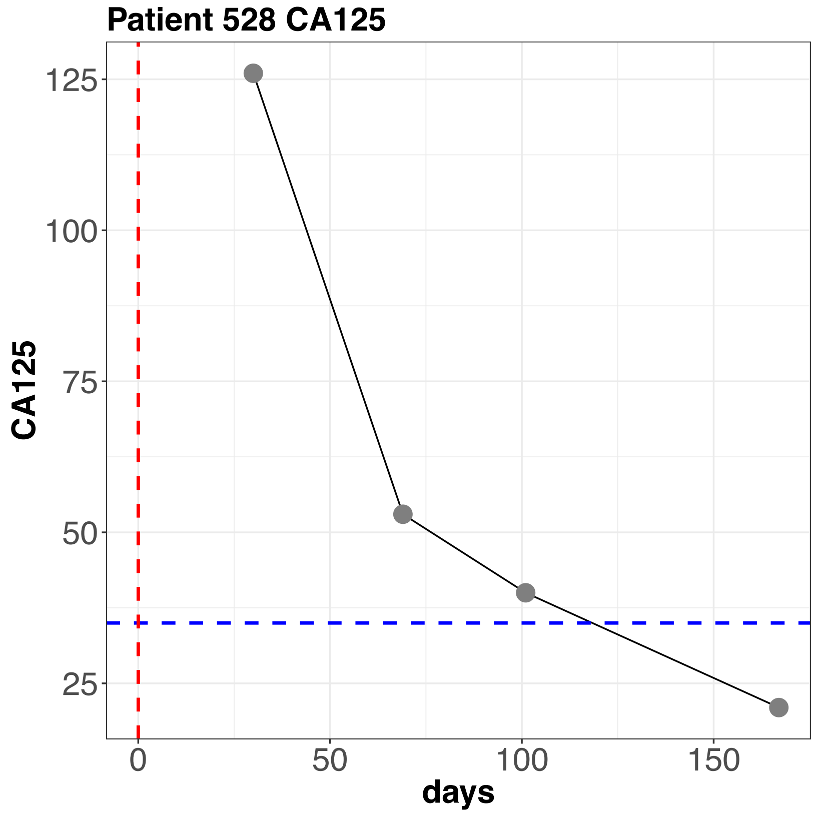
Patient 528 CA125
CA125
125
100
75
50
25
0
50
100
150
days

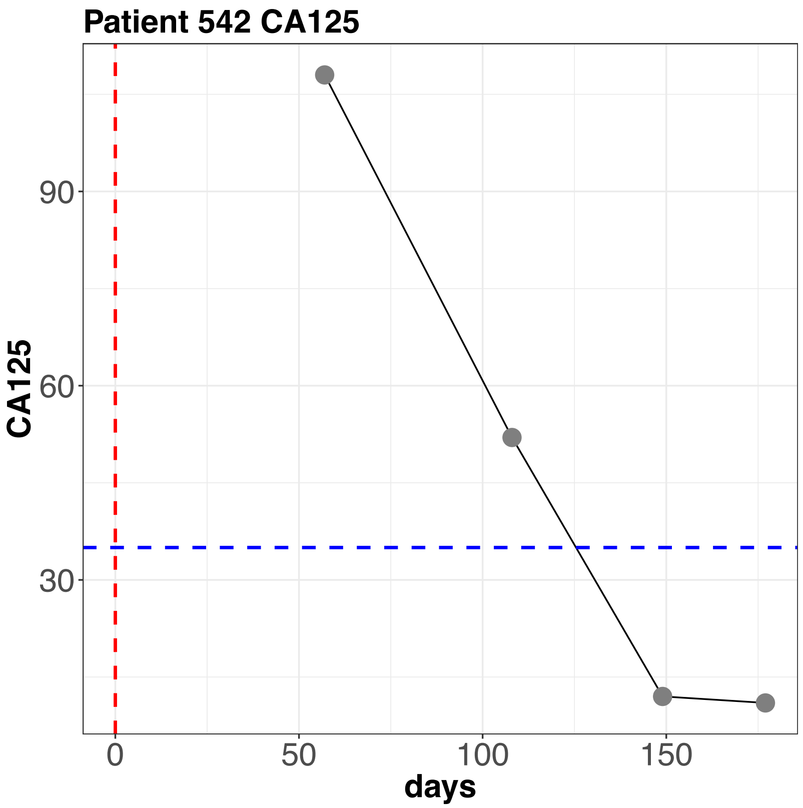
Patient 542 CA125
CA125
90
60
30
0
50
100
150
days

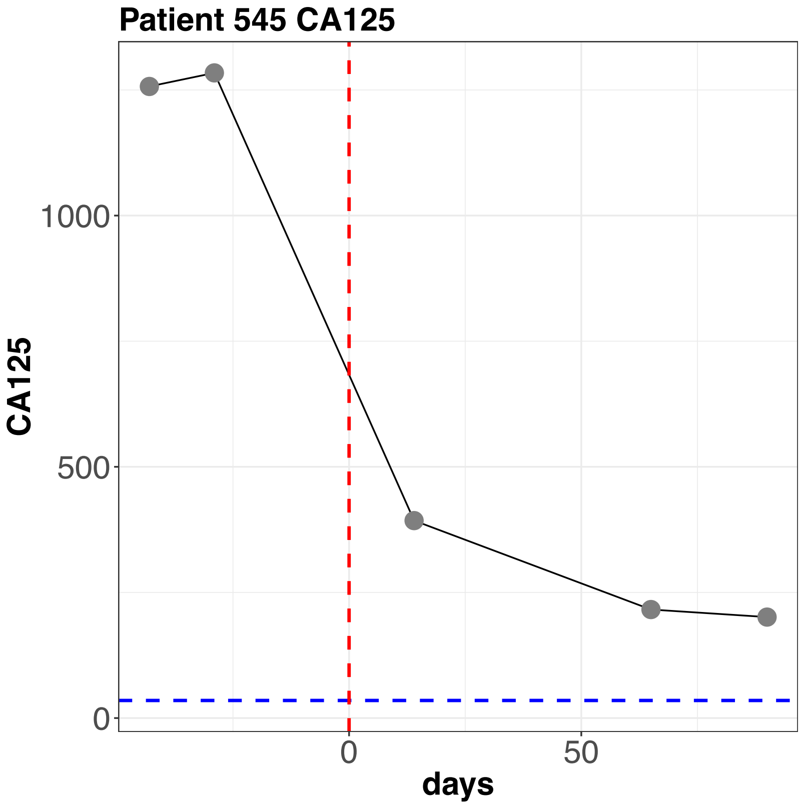
Patient 545 CA125
CA125
1000
500
0
0
50
days

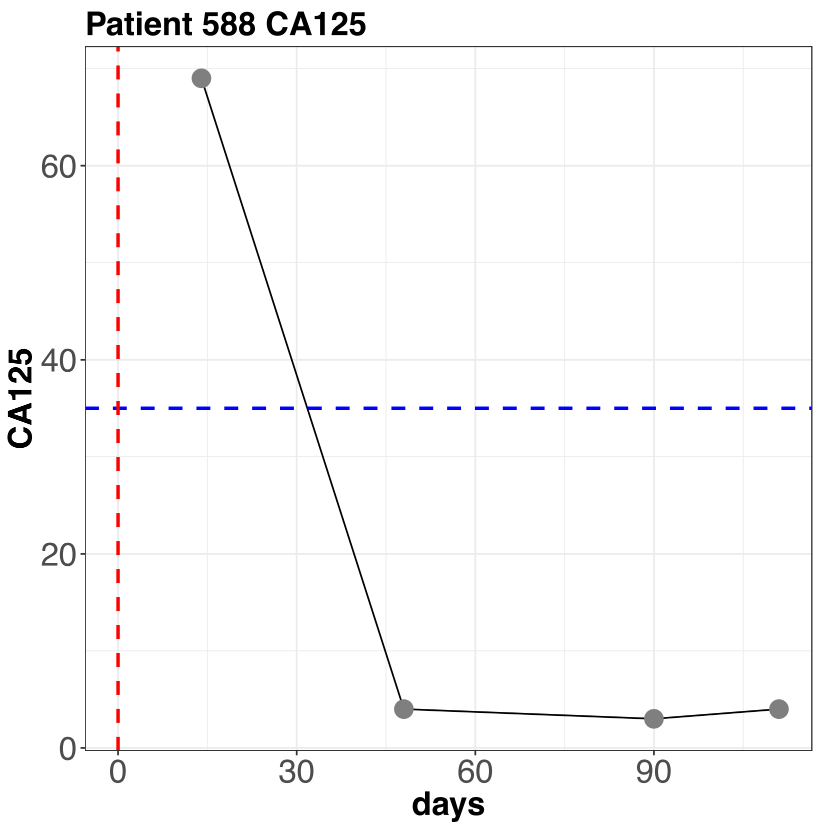

Patient 588 CA125
CA125
0
20
40
60
0
30
60
90
days

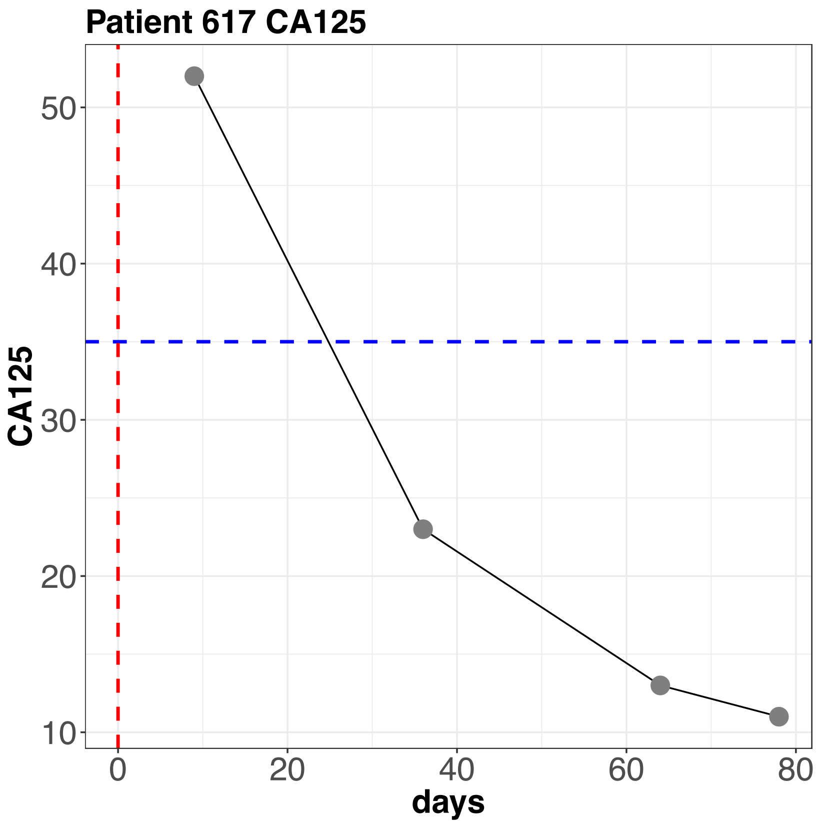

Patient 617 CA125
CA125
10
20
30
40
50
0
20
40
60
80
days

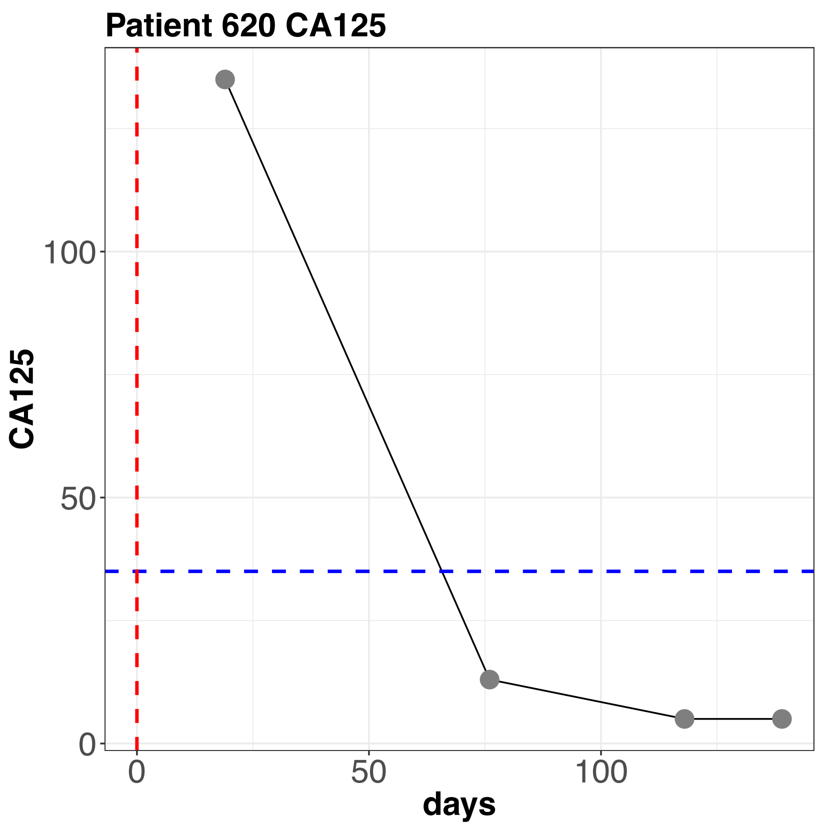
Patient 620 CA125
CA125
100
50
0
0
50
100
days

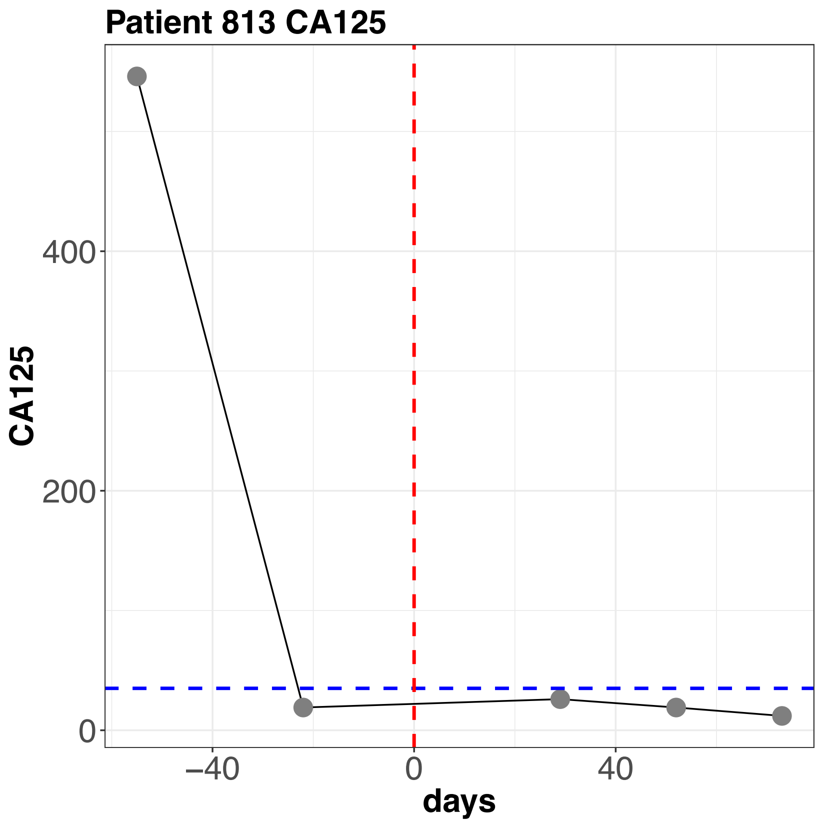
Patient 813 CA125
CA125
400
200
0
−40
0
40
days

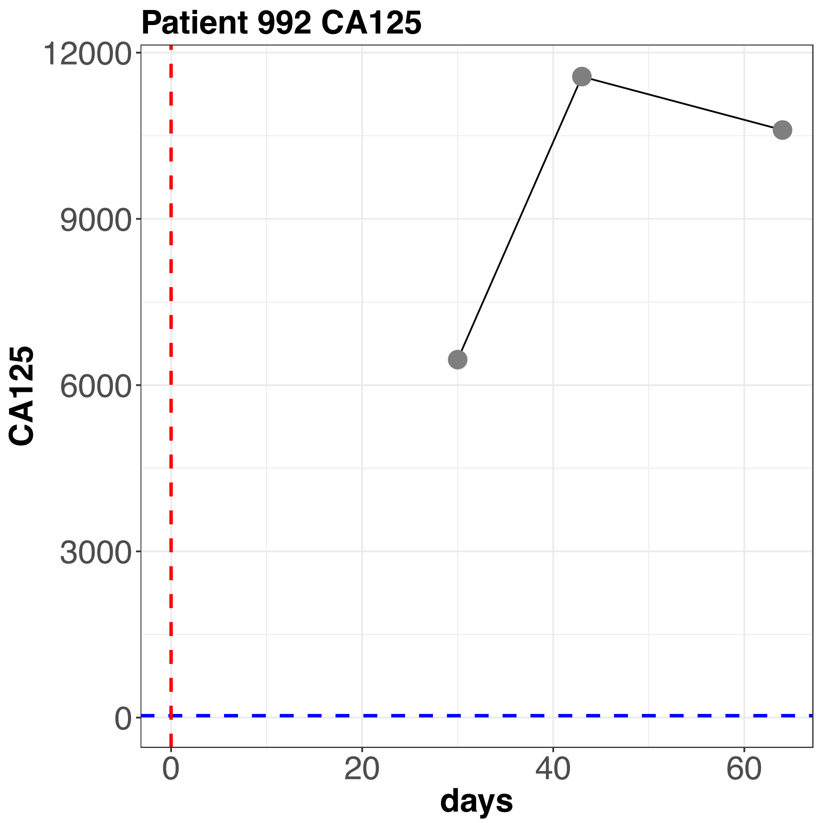

Patient 992 CA125
12000
9000
6000
3000
0
CA125
0
20
40
60
days

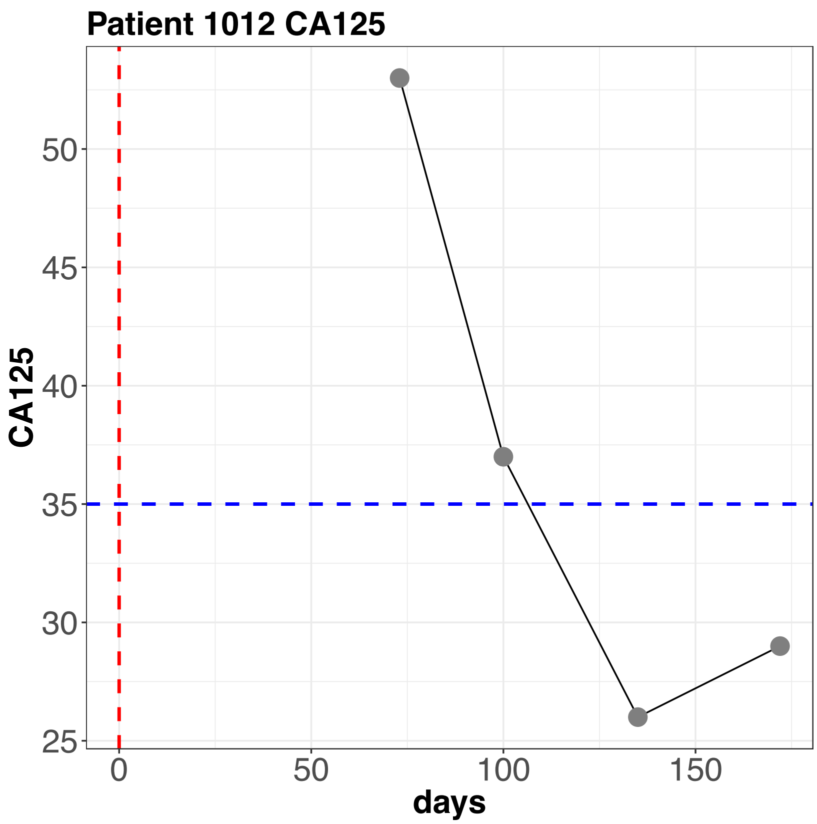

Patient 1012 CA125
50
45
40
35
30
25
CA125
0
50
100
150
days

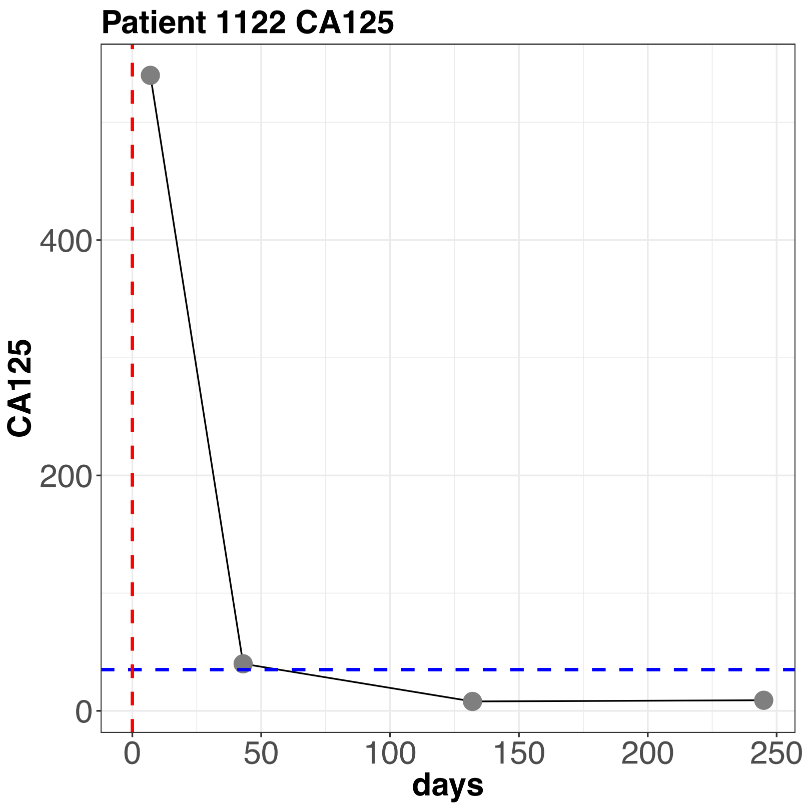

Patient 1122 CA125
CA125
400
200
0
0
50
100
150
200
250
days

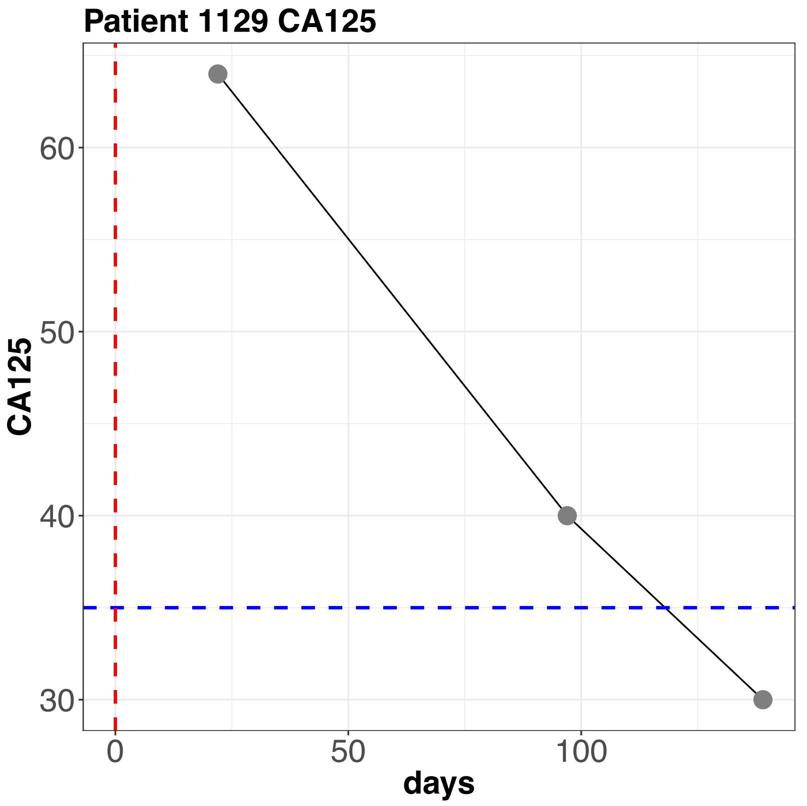

Patient 1129 CA125
CA125
60
50
40
30
0
50
100
days

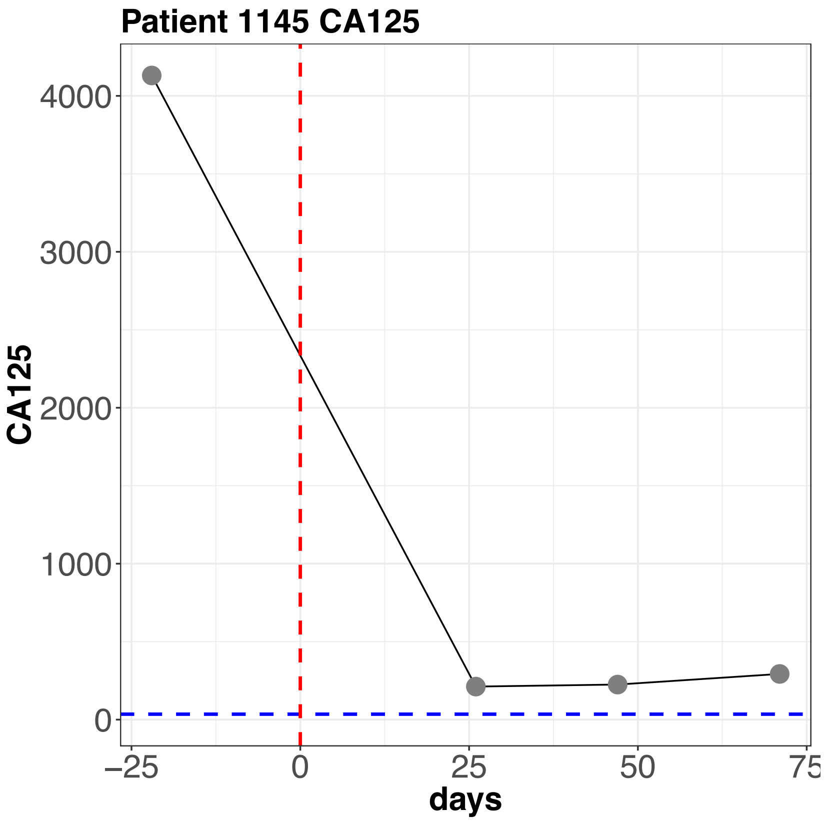
Patient 1145 CA125
CA125
4000
3000
2000
1000
0
−25
0
25
50
75
days

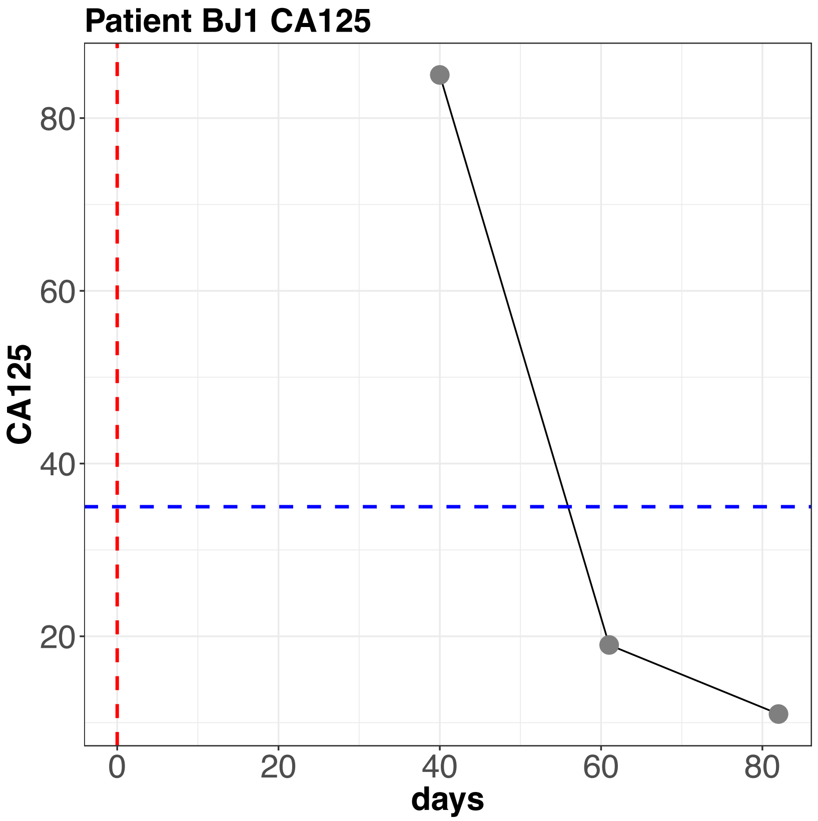
Patient BJ1 CA125
CA125
80
60
40
20
0
20
40
60
80
days

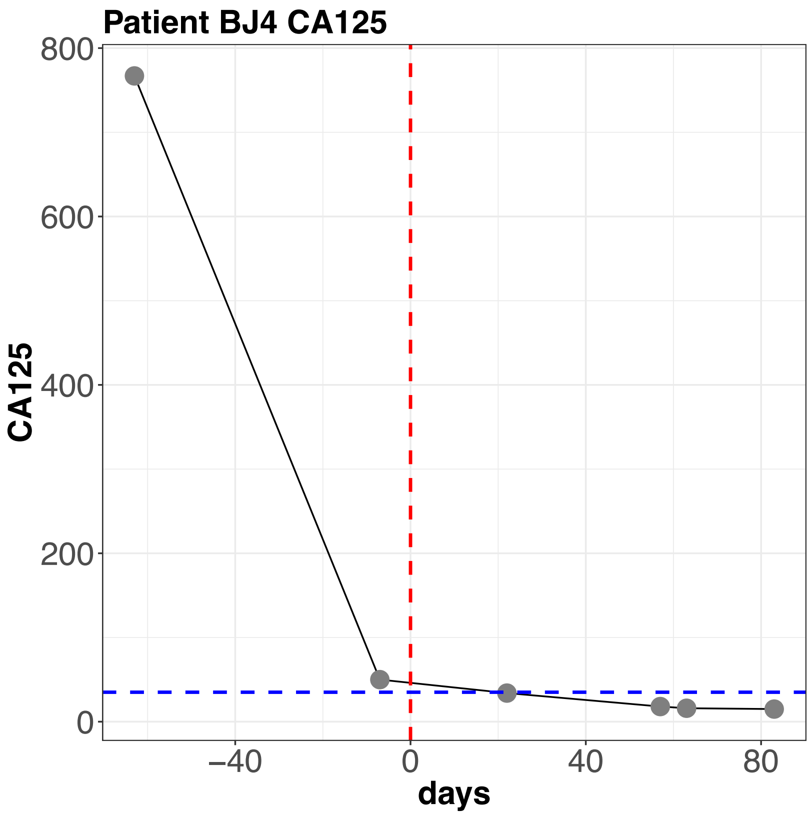

Patient BJ4 CA125
CA125
800
600
400
200
0
−40
0
40
80
days

Supplementary Table S1. Number and types of cancer patients responding to gemcitabine or 5-fluorouracil chemotherapeutic treatments.

Cancer Type	Drug	No. Responsive	No. Not Responsive	Total No. Samples
bladder urothelial carcinoma	Gemcitabine	4	4	8
breast invasive carcinoma	Gemcitabine	1	1	2
cervical squamous cell carcinoma	Gemcitabine	1	0	1
cholangiocarcinoma	Gemcitabine	3	1	4
head/neck squamous cell carcinoma	Gemcitabine	0	1	1
liver hepatocellular carcinoma	Gemcitabine	0	2	2
lung adenocarcinoma	Gemcitabine	1	2	3
lung squamous cell carcinoma	Gemcitabine	1	2	3
pancreatic adenocarcinoma	Gemcitabine	24	33	57
phenochromocytoma/paraganglioma	Gemcitabine	0	1	1
sarcoma	Gemcitabine	1	5	6
skin cutaneous melanoma	Gemcitabine	0	2	2
testicular germ cell tumors	Gemcitabine	1	0	1
uterine corpus endometrial carcinoma	Gemcitabine	0	1	1
**Sub-total**	**Gemcitabine**	**37**	**55**	**92**
colon adenocarcinoma	5-fluorouracil	2	2	4
esophageal carcinoma	5-fluorouracil	2	1	3
pancreatic adenocarcinoma	5-fluorouracil	3	6	9
rectal adenocarcinoma	5-fluorouracil	9	0	9
stomach adenocarcinoma	5-fluorouracil	19	16	35
**Sub-total**	**5-fluorouracil**	**35**	**25**	**60**

Supplementary Table S2. Predicted and observed responses of 60 cancer patients treated with 5-fluorouricil and 92 cancer patients treated with gemcitabine.
(R = responder; NR = non-responder; TN = true negative; FP = false positive; TP = true positive; FN = false negative)

Patient ID	Drug	OBSERVED RESPONSE	PREDICTED RESPONSE
F1	Fluorouracil	NR	NR(TN)
F2	Fluorouracil	NR	NR(TN)
F3	Fluorouracil	NR	NR(TN)
F4	Fluorouracil	NR	R(FP)
F5	Fluorouracil	NR	R(FP)
F6	Fluorouracil	NR	NR(TN)
F7	Fluorouracil	NR	NR(TN)
F8	Fluorouracil	NR	NR(TN)
F9	Fluorouracil	NR	NR(TN)
F10	Fluorouracil	NR	NR(TN)
F11	Fluorouracil	NR	NR(TN)

F12	Fluorouracil	NR	NR(TN)
F13	Fluorouracil	NR	R(FP)
F14	Fluorouracil	NR	NR(TN)
F15	Fluorouracil	NR	NR(TN)
F16	Fluorouracil	NR	NR(TN)
F17	Fluorouracil	NR	R(FP)
F18	Fluorouracil	NR	NR(TN)
F19	Fluorouracil	NR	NR(TN)
F20	Fluorouracil	NR	R(FP)
F21	Fluorouracil	NR	NR(TN)
F22	Fluorouracil	NR	R(FP)
F23	Fluorouracil	NR	NR(TN)
F24	Fluorouracil	NR	NR(TN)
F25	Fluorouracil	NR	NR(TN)
F26	Fluorouracil	R	R(TP)
F27	Fluorouracil	R	R(TP)
F28	Fluorouracil	R	R(TP)
F29	Fluorouracil	R	R(TP)
F30	Fluorouracil	R	R(TP)
F31	Fluorouracil	R	R(TP)
F32	Fluorouracil	R	NR(FN)
F33	Fluorouracil	R	R(TP)
F34	Fluorouracil	R	NR(FN)
F35	Fluorouracil	R	R(TP)
F36	Fluorouracil	R	R(TP)
F37	Fluorouracil	R	R(TP)
F38	Fluorouracil	R	NR(FN)
F39	Fluorouracil	R	R(TP)
F40	Fluorouracil	R	R(TP)
F41	Fluorouracil	R	R(TP)
F42	Fluorouracil	R	R(TP)
F43	Fluorouracil	R	R(TP)
F44	Fluorouracil	R	R(TP)
F45	Fluorouracil	R	R(TP)
F46	Fluorouracil	R	R(TP)
F47	Fluorouracil	R	R(TP)
F48	Fluorouracil	R	R(TP)
F49	Fluorouracil	R	R(TP)
F50	Fluorouracil	R	R(TP)
F51	Fluorouracil	R	R(TP)
F52	Fluorouracil	R	NR(FN)
F53	Fluorouracil	R	R(TP)
F54	Fluorouracil	R	R(TP)

F55	Fluorouracil	R	NR(FN)
F56	Fluorouracil	R	R(TP)
F57	Fluorouracil	R	R(TP)
F58	Fluorouracil	R	R(TP)
F59	Fluorouracil	R	R(TP)
F60	Fluorouracil	R	R(TP)
Totals:			30TP,19TN,6FP,5FN

Patient ID	Drug	OBSERVED RESPONSE	PREDICTED RESPONSE
G1	Gemcitabine	NR	NR(TN)
G2	Gemcitabine	NR	NR(TN)
G3	Gemcitabine	NR	NR(TN)
G4	Gemcitabine	NR	R(FP)
G5	Gemcitabine	NR	NR(TN)
G6	Gemcitabine	NR	R(FP)
G7	Gemcitabine	NR	NR(TN)
G8	Gemcitabine	NR	NR(TN)
G9	Gemcitabine	NR	NR(TN)
G10	Gemcitabine	NR	NR(TN)
G11	Gemcitabine	NR	NR(TN)
G12	Gemcitabine	NR	NR(TN)
G13	Gemcitabine	NR	R(FP)
G14	Gemcitabine	NR	NR(TN)
G15	Gemcitabine	NR	NR(TN)
G16	Gemcitabine	NR	NR(TN)
G17	Gemcitabine	NR	NR(TN)
G18	Gemcitabine	NR	R(FP)
G19	Gemcitabine	NR	NR(TN)
G20	Gemcitabine	NR	NR(TN)
G21	Gemcitabine	NR	NR(TN)
G22	Gemcitabine	NR	R(FP)
G23	Gemcitabine	NR	NR(TN)
G24	Gemcitabine	NR	NR(TN)
G25	Gemcitabine	NR	NR(TN)
G26	Gemcitabine	NR	NR(TN)
G27	Gemcitabine	NR	NR(TN)
G28	Gemcitabine	NR	R(FP)
G29	Gemcitabine	NR	NR(TN)
G30	Gemcitabine	NR	NR(TN)
G31	Gemcitabine	NR	NR(TN)
G32	Gemcitabine	NR	NR(TN)
G33	Gemcitabine	NR	NR(TN)

G34	Gemcitabine	NR	NR(TN)
G35	Gemcitabine	NR	NR(TN)
G36	Gemcitabine	NR	NR(TN)
G37	Gemcitabine	NR	NR(TN)
G38	Gemcitabine	NR	R(FP)
G39	Gemcitabine	NR	NR(TN)
G40	Gemcitabine	NR	NR(TN)
G41	Gemcitabine	NR	NR(TN)
G42	Gemcitabine	NR	NR(TN)
G43	Gemcitabine	NR	NR(TN)
G44	Gemcitabine	NR	R(FP)
G45	Gemcitabine	NR	NR(TN)
G46	Gemcitabine	NR	NR(TN)
G47	Gemcitabine	NR	NR(TN)
G48	Gemcitabine	NR	NR(TN)
G49	Gemcitabine	NR	NR(TN)
G50	Gemcitabine	NR	NR(TN)
G51	Gemcitabine	NR	NR(TN)
G52	Gemcitabine	NR	NR(TN)
G53	Gemcitabine	NR	NR(TN)
G54	Gemcitabine	NR	NR(TN)
G55	Gemcitabine	NR	NR(TN)
G56	Gemcitabine	R	R(TP)
G57	Gemcitabine	R	NR(FN)
G58	Gemcitabine	R	R(TP)
G59	Gemcitabine	R	R(TP)
G60	Gemcitabine	R	R(TP)
G61	Gemcitabine	R	R(TP)
G62	Gemcitabine	R	R(TP)
G63	Gemcitabine	R	NR(FN)
G64	Gemcitabine	R	R(TP)
G65	Gemcitabine	R	R(TP)
G66	Gemcitabine	R	R(TP)
G67	Gemcitabine	R	R(TP)
G68	Gemcitabine	R	R(TP)
G69	Gemcitabine	R	R(TP)
G70	Gemcitabine	R	R(TP)
G71	Gemcitabine	R	R(TP)
G72	Gemcitabine	R	R(TP)
G73	Gemcitabine	R	R(TP)
G74	Gemcitabine	R	R(TP)
G75	Gemcitabine	R	R(TP)
G76	Gemcitabine	R	NR(FN)

G77	Gemcitabine	R	R(TP)
G78	Gemcitabine	R	R(TP)
G79	Gemcitabine	R	R(TP)
G80	Gemcitabine	R	NR(FN)
G81	Gemcitabine	R	NR(FN)
G82	Gemcitabine	R	R(TP)
G83	Gemcitabine	R	R(TP)
G84	Gemcitabine	R	R(TP)
G85	Gemcitabine	R	R(TP)
G86	Gemcitabine	R	R(TP)
G87	Gemcitabine	R	NR(FN)
G88	Gemcitabine	R	NR(FN)
G89	Gemcitabine	R	R(TP)
G90	Gemcitabine	R	NR(FN)
G91	Gemcitabine	R	NR(FN)
G92	Gemcitabine	R	R(TP)
Totals:			28TP,47TN,8FP,9FN

For Supplementary Table S3 see additional data file (xslx).

Supplementary Table S4. Genes associated with optimal predictive accuracy for gemcitabine (81 genes) and 5-fluorouracil (31 genes).

5-Fluorouracil

ensembl_gene_id	hgnc_ gene symbol
ENSG00000003096.12	KLHL13
ENSG00000086848.13	ALG9
ENSG00000117475.12	BLZF1
ENSG00000120029.11	C10orf76
ENSG00000120549.14	KIAA1217
ENSG00000135951.13	TSGA10
ENSG00000173338.11	KCNK7
ENSG00000178074.5	C2orf69
ENSG00000182950.2	ODF3L1
ENSG00000196083.8	IL1RAP
ENSG00000207698.1	MIR32
ENSG00000207895.1	PPIAP29
ENSG00000214975.4	not functionally annotated
ENSG00000227680.1	not functionally annotated
ENSG00000228031.2	not functionally annotated
ENSG00000230269.5	not functionally annotated
ENSG00000232813.1	TMEM92-AS1
ENSG00000236360.2	RNU6-1231P
ENSG00000251179.1	not functionally annotated

ENSG00000252994.1	not functionally annotated
ENSG00000254850.2	not functionally annotated
ENSG00000257264.3	HMGB3P32
ENSG00000260671.2	not functionally annotated
ENSG00000260828.1	not functionally annotated
ENSG00000260920.2	not functionally annotated
ENSG00000266176.1	not functionally annotated
ENSG00000266821.1	not functionally annotated
ENSG00000267633.1	not functionally annotated
ENSG00000271394.1	not functionally annotated
ENSG00000279075.1	not functionally annotated
ENSG00000279144.1	not functionally annotated

Gemcitabine

ensembl_gene_id.	hgnc_ gene symbol
ENSG00000085265.9	FCN1
ENSG00000101935.8	AMMECR1
ENSG00000106123.10	EPHB6
ENSG00000111863.11	ADTRP
ENSG00000113739.9	STC2
ENSG00000131355.13	ADGRE3
ENSG00000131473.15	ACLY
ENSG00000136960.11	ENPP2
ENSG00000138134.10	STAMBPL1
ENSG00000139351.13	SYCP3
ENSG00000146109.4	ABT1
ENSG00000147571.4	CRH
ENSG00000160223.15	ICOSLG
ENSG00000165102.13	HGSNAT
ENSG00000165682.13	CLEC1B
ENSG00000170703.14	TTLL6
ENSG00000172900.10	not functionally annotated
ENSG00000173389.14	IQCF1
ENSG00000180720.7	CHRM4
ENSG00000181374.6	CCL13
ENSG00000183019.6	MCEMP1
ENSG00000185220.10	PGBD2
ENSG00000187616.4	MYMK
ENSG00000188477.11	LYPD2
ENSG00000197353.3	ELANE
ENSG00000197561.5	COL4A6
ENSG00000197565.14	HOXA4
ENSG00000197576.12	not functionally annotated
ENSG00000200591.1	RNY4P17

ENSG00000201818.1	not functionally annotated
ENSG00000202279.1	SNORA70B
ENSG00000206937.1	RNU6-790P
ENSG00000207208.1	KLHL23
ENSG00000213160.8	SELENOWP1
ENSG00000215900.4	RNU6-584P
ENSG00000222282.1	SNORA79B
ENSG00000222489.1	not functionally annotated
ENSG00000223343.1	HMGB1P9
ENSG00000224159.1	SLC9A3-AS1
ENSG00000225138.6	not functionally annotated
ENSG00000225744.1	not functionally annotated
ENSG00000228157.4	AMD1P1
ENSG00000228339.1	KHSRPP1
ENSG00000229835.2	not functionally annotated
ENSG00000230534.5	not functionally annotated
ENSG00000232615.4	not functionally annotated
ENSG00000233514.1	not functionally annotated
ENSG00000239455.1	PPIL3
ENSG00000240344.7	RN7SL809P
ENSG00000241217.3	GAPDHP47
ENSG00000241781.2	GUCY2GP
ENSG00000243033.2	not functionally annotated
ENSG00000243316.5	RN7SL762P
ENSG00000243961.2	SNRPCP3
ENSG00000244308.2	RN7SKP197
ENSG00000244668.1	IGLVI-70
ENSG00000252886.1	IGLV3-30
ENSG00000253239.1	not functionally annotated
ENSG00000253329.1	IGHV1-17
ENSG00000253475.1	HSPD1P2
ENSG00000254046.1	not functionally annotated
ENSG00000254543.1	not functionally annotated
ENSG00000256533.1	not functionally annotated
ENSG00000256948.1	LINC01169
ENSG00000258520.1	not functionally annotated
ENSG00000259471.1	not functionally annotated
ENSG00000259871.1	COX6CP1
ENSG00000259931.2	not functionally annotated
ENSG00000260318.1	not functionally annotated
ENSG00000260776.4	TXNIP
ENSG00000261212.1	not functionally annotated
ENSG00000265212.2	not functionally annotated
ENSG00000265972.4	not functionally annotated

ENSG00000266924.1	LINC01081
ENSG00000267293.1	not functionally annotated
ENSG00000268036.1	YWHAQP7
ENSG00000268754.1	not functionally annotated
ENSG00000269495.1	not functionally annotated
ENSG00000271142.1	not functionally annotated
ENSG00000274979.1	not functionally annotated
ENSG00000278532.1	not functionally annotated

Supplementary Table S5. Clinical stage, grade and type of 23 ovarian cancer patient tumors included in this study.

Patient ID	age at time of surgery	histopathology of tumor	stage	grade
229	58	serous papillary	IIIc	3
242	63	serous papillary	IIIb	3
272	83	adenocarcinoma	IIIb	2/3
286	52	serous papillary	IIIc	2/3
317	59	serous papillary	Ic	3
336	63	serous papillary	Ic	3
367	56	serous papillary	II	3
413	49	serous papillary	IIb	3
489	48	serous papillary	IV	3
528	66	serous papillary	IIIc	3
542	61	serous papillary	IV	3
545	74	MMMT	IIIc	3
588	71	serous papillary	IIIc	2/3
617	64	serous papillary	IIIc	2/3
620	62	serous papillary	III/IV	3
813	56	adenocarcinoma	III	1/2
992	73	serous papillary	IIIc	3
1012	75	serous papillary	IIIc	3
1122	65	serous papillary	IIIc	3
1129	65	serous papillary	IIIc	3
1145	41	serous papillary	IIIc	3
BJ1	40	serous papillary	III	3
BJ4	56	serous papillary	IIIc	3
